# Coupling solid and fluid stresses with brain tumour growth and white matter tract deformations in a neuroimaging-informed model

**DOI:** 10.1007/s10237-022-01602-4

**Published:** 2022-07-30

**Authors:** Giulio Lucci, Abramo Agosti, Pasquale Ciarletta, Chiara Giverso

**Affiliations:** 1 Department of Mathematical Sciences “G.L. Lagrange”, Politecnico di Torino, Corso Duca degli Abruzzi 24, 10129 Turin, Italy; 2 Department of Mathematics, University of Pavia, Via Ferrata 5, 27100 Pavia, Italy; 3 MOX-Politecnico di Milano, Piazza Leonardo da Vinci 23, 20133 Milan, Italy

**Keywords:** Brain tumour growth, Cancer modelling, Continuum Mechanics, Mixture theory, Nonlinear elasticity, Finite element method, 74B20, 74L15, 92C50

## Abstract

Brain tumours are among the deadliest types of cancer, since they display a strong ability to invade the surrounding tissues and an extensive resistance to common therapeutic treatments. It is therefore important to reproduce the heterogeneity of brain microstructure through mathematical and computational models, that can provide powerful instruments to investigate cancer progression. However, only a few models include a proper mechanical and constitutive description of brain tissue, which instead may be relevant to predict the progression of the pathology and to analyse the reorganization of healthy tissues occurring during tumour growth and, possibly, after surgical resection. Motivated by the need to enrich the description of brain cancer growth through mechanics, in this paper we present a mathematical multiphase model that explicitly includes brain hyperelasticity. We find that our mechanical description allows to evaluate the impact of the growing tumour mass on the surrounding healthy tissue, quantifying the displacements, deformations, and stresses induced by its proliferation. At the same time, the knowledge of the mechanical variables may be used to model the stress-induced inhibition of growth, as well as to properly modify the preferential directions of white matter tracts as a consequence of deformations caused by the tumour. Finally, the simulations of our model are implemented in a personalized framework, which allows to incorporate the realistic brain geometry, the patient-specific diffusion and permeability tensors reconstructed from imaging data and to modify them as a consequence of the mechanical deformation due to cancer growth.

**Supplementary Information:**

The online version contains supplementary material available at 10.1007/s10237-022-01602-4.

## Introduction

The vast majority of brain tumours, such as gliomas (the most frequent ones, arising from glial cells), grow along the white matter fibre tracts or along vessels, following the physical structures in the extracellular environment. Therefore, they show an irregular evolution and their final shapes can substantially differ from the spherical one (Hatzikirou et al. 2005). As far as treatment is concerned, brain cancers are extensively resistant to therapies, especially chemotherapy (Carlson 2012), and a complete treatment usually starts with surgery and removal of as much of tumour mass as possible. However, since the tumour might infiltrate and damage eloquent areas and structures of the brain, it is generally difficult and sometimes impossible to fully resect the cancerous mass (Hatzikirou et al. 2005).

For all these reasons, there is a critical need to understand and replicate the biological complexity of the brain, in order to predict brain tumour evolution and forecast the possibly injured areas. To this end, mathematical and computational models can provide powerful instruments for investigating cancer progression, especially in those cases that are particularly difficult to be treated with current therapeutic protocols, such as glioblastoma multiforme (GBM), one of the most aggressive and malignant brain tumours, as well as the most common ones (Young et al. 2015; Stupp and Hegi 2009; Ostrom et al. 2014). In the last decades, several mathematical models of brain tumour growth have been proposed, with the purpose of providing a better understanding of the phenomenon while speeding up the research process through the use of computer simulations. At the microscopic and mesoscopic level, discrete computational approaches such as cellular automata or agent-based models represent useful tools to explore invasive migration, phenotypic plasticity, and early growth of brain tumours (Kansal et al. 2000; Khain et al. 2011; Aubert et al. 2008; Tektonidis et al. 2011; Mansury et al. 2002). At the macroscopic scale, continuum models are more suitable to describe brain tumour cell motility and spatial dynamics through advection–reaction–diffusion equations (Murray 2003; Tracqui et al. 1995; Swanson et al. 2000, 2002a, b, 2003a, b; Stein et al. 2007; Swan et al. 2018) or kinetic equations (Painter and Hillen 2013), including characteristics of brain tissue such as heterogeneity and anisotropy coming from medical imaging (Jbabdi et al. 2005; Painter and Hillen 2013; Swan et al. 2018). Another recent approach for brain tumour modelling employs diffuse interface multiphase models of Cahn–Hilliard type (Cahn and Hilliard 1958), introducing a fourth-order nonlinear advection–reaction–diffusion equation, which has been successfully applied to describe the evolution of GBM (Colombo et al. 2015; Agosti et al. 2018a, b).

Despite the ability of these models to qualitatively capture some peculiar features of the growth of a brain tumour, they do not account for some important mechanical aspects, such as the influence of the stress exerted by the healthy tissue on the tumour mass and vice versa. Indeed, not only the growth of the tumour might be limited by the surrounding tissue, as observed in many biological experiments in vitro (Cheng et al. 2009; Helmlinger et al. 1997; Montel et al. 2012; Delarue et al. 2014), but also the presence of a neoplasm may be a critical clinical issue inside the healthy peripheral tissue subject to unnatural displacements. Although tumour growth can adversely impact the health of any hosting organ, this is especially devastating in the brain. As a matter of fact, compared with extracranial organs, the brain is unique because of its physical confinement due to the skull fixed volume, which can further amplify mechanical force effects. Furthermore, brain functions might be corrupted by mechanical forces: the tumour growth-induced deformation and compression is believed to be a major cause of the neurological clinical symptoms and severe disabilities seen in patients with brain cancer, and represents a negative prognostic factor (Gamburg et al. 2000; Kalli et al. 2019; Steed et al. 2018). The identification of the importance of mechanical cues and their potential regulatory roles in the development and maintenance of neuronal structures (Motz et al. 2021) has led to the definition of a new field of research, named “neuromechanobiology”, dealing with the effects of mechanical forces on normal neurophysiology and on neurological disorders (Motz et al. 2021; Bouwen et al. 2018; Amidei and Kushner 2015; Bryniarska-Kubiak et al. 2021). In this regard, understanding how injured and healthy brain fibre tracts deform and re-distribute in response to the growing tumour mass is a fundamental issue. In particular, mechanical forces could be exerted either by the tumour-associated oedema or by the solid components of the malignant tissue, such as cells and extracellular matrix (Seano et al. 2019). The latter is often referred to as *solid stress* or *mass-effect* and its origin and biological consequences are still poorly understood, with respect to the fluid pressure associated with oedema, a well-known mechanical abnormality in brain tumours (Chauhan et al. 2014; Goriely et al. 2015; Jain et al. 2014; Seano et al. 2019). Although, recently, the origin and neurological effects of the solid stress have gained attention, details of their quantification in vitro and their biological impact on the physiology of the healthy brain surrounding the tumour remain unknown (Seano et al. 2019; Kalli et al. 2019). Concerning these latter aspects, the tumour-generated solid stress consistently distorts the micro-anatomy of the neighbouring brain tissue and it compresses the blood vessels, generating a vascular collapse. Consequently, there is a reduction of peritumoural vascular perfusion, contributing to intratumoral hypoxia, inducing neuronal loss, and hindering the delivery and efficacy of anti-cancer therapies (Chauhan et al. 2014; Padera et al. 2004; Seano et al. 2019; Nia et al. 2020). The tumour-induced deformation of the healthy tissue is reflected by the distortions macroscopically observed in radiological exams (e.g. the shift of the cranial midline), which is more evident around “nodular” tumours, with well-defined margins, with respect to “infiltrative” tumours, that invade into the surrounding tissue as individual cells (Abler et al. 2019; Steed et al. 2018; Gamburg et al. 2000; Nia et al. 2020). Since tumours of similar imaging volumes have been observed to give rise to different amounts and distributions of solid stresses (Steed et al. 2018; Nia et al. 2020), it is relevant to evaluate deformations, stresses, and displacement caused by their progression, in order to properly capture the correct area of the brain influenced by the cancer.

In the light of all these observations, from the mathematical modelling point of view, it is fundamental to account for a mechanical and constitutive description of brain tissue and tumour, that has been neglected in the vast majority of previous brain cancer growth models. Some attempts to include the mechanical aspects have been done  by Clatz et al. (2005), coupling a reaction–diffusion model, for simulating the invasion of the tumour in the brain parenchyma, with a linear elastic brain constitutive equation, to describe the mechanical interaction with the invaded tissue. Then, Lang et al. (2015) developed a model for propagation of damage and oedema in brain tissue using an iterative approach and Continuum Mechanics. Ehlers and Wagner (2015) proposed a more elaborated model for brain tissues and drug delivery, assuming the presence of three phases (an hyperelastic and mechanically anisotropic solid skeleton, the blood, and the interstitial fluid), but since they do not take into account the growth of the solid phase, their model is not suitable to describe tumour progression. Indeed, in the development of a mathematical model that includes mechanics of a growing mass, some non-trivial difficulties arise: cells duplicate and die, the environment is continuously modified and remodelled as a result of tumour growth, and when dealing with solid tumours it is not clear which reference configuration should be used to measure deformations, since the material is constantly changing (Preziosi and Tosin 2009; Giverso et al. 2015; Ambrosi et al. 2017; Di Stefano et al. 2018; Mascheroni et al. 2018; Grillo et al. 2019; Ehlers et al. 2022). In the context of tumour growth and biological applications, this problem was tackled in Ambrosi and Mollica (2002), Ambrosi and Mollica (2004) by applying the concept of “evolving natural configurations” (Rajagopal 1995; Lubarda 2004; DiCarlo and Quiligotti 2002), which—loosely speaking—consists in splitting the evolution in pure elastic deformations and deformations subsequent to growth. Some recent works on macroscopic models for brain tumour growth have been developed using this framework (Mascheroni et al. 2016, 2018; Angeli and Stylianopoulos 2016; Angeli et al. 2018; Ehlers et al. 2022). Nevertheless, in these latter works, the patient-specific anisotropy is not included and the effect of fibre deformations subsequent to tumour growth, as well as the impact on the diffusion of chemical species and on the motion of cells, have not been investigated.

Therefore, stimulated by the need to elaborate a more refined description of brain tumour mechanical impact, in this work we develop a mathematical model for cancer growth and proliferation which includes brain hyperelasticity, in order to evaluate the effects of structural changes in the white matter and the nonlinear elastic deformations of brain tissue. In particular, we propose a macroscopic model based on finite deformations and Continuum Mechanics: we treat both the tumour and the surrounding tissue as saturated biphasic mixtures, composed by a hyperelastic solid phase and an ideal fluid phase. Moreover, we consider the tumour as separated from the host tissue through a smooth regularization of an indicator function. In order to distinguish the elastic deformations from the inelastic distortions caused by growth, we employ a multiplicative decomposition of the deformation gradient (Ambrosi and Mollica 2002; DiCarlo and Quiligotti 2002). To test the validity of our model as a proof-of-concept, we perform simulations on a realistic brain geometry, reconstructed from magnetic resonance imaging (MRI) and diffusion tensor imaging (DTI) data. This allows us to introduce patient-specific brain tissue anisotropy in our model, by reconstructing spatially dependent diffusion and permeability tensors from medical images. Furthermore, after the reconstruction of the starting clinical data, we take advantage of the mechanical description to progressively modify the mentioned tensors in time. Indeed, the knowledge of the mechanical tumour impact allows to properly adjust the preferential directions for diffusion and fluid motion, following the deformation induced by the mass onto the surrounding tissue.

In detail, the paper is organized as follows. In Sect. 2 we derive our mechanical model and its governing equations, also providing an estimate for all the relevant parameters. Then, in Sect. 3 we discuss the details concerning the numerical implementation and computational mesh reconstruction from imaging data. Section 4 is dedicated to the presentation of numerical simulation results. Finally, in Sect. 5 we summarize the main features of the work and present possible directions for further research.

## Theory and calculations

In this section, we derive a continuous mechanical theory for modelling the macroscopic brain tumour growth using a multiphase approach and the evolving natural configurations framework. We consider both the healthy and the tumour brain tissue as saturated domains comprising two distinct phases, which represent the cell population (labelled with subscript “$\text {s}$”) and the interstitial fluid (labelled with subscript “$\ell$”). Moreover, the cancer and the host tissue are localized in different regions, denoted by a smooth approximation of an indicator function.

### Mathematical model

#### Kinematics and growth framework

At a given time *t*, we consider the current configuration of the brain as a three-dimensional domain $\Omega (t)$ and denote by $\Omega _{\mathrm{t}}(t)$ the subregion occupied by the growing tumour, while $\Omega _{\mathrm{h}}(t)$ stands for the subregion occupied by the healthy tissue, with $\Omega (t)=\Omega _{\mathrm{t}}(t) \cup \Omega _{\mathrm{h}}(t)$. In particular, the tumour region is identified by a smooth approximation of the indicator function $\chi _{{\Omega _{\mathrm{t}}(t)}}$ of the cancerous domain, which moves at the velocity of diseased cells. The smoothness of $\chi _{{\Omega _{\mathrm{t}}(t)}}$ allows to account for regions of coexistence of tumour cells and healthy cells near the cancer mass. Such a description is appropriate to describe solid and low-grade brain tumours, that are mostly localized and characterized by a superposition of healthy and diseased tissues only around the principal mass, without colonies of growing and invading cancer cells detached from the tumour bulk. In particular, we identify the tumour domain with the upper level set $\Omega _{\mathrm{t}}(t) = \{ \mathbf {x} \in \mathbb {R}^3 \, : \, \chi _{{\Omega _{\mathrm{t}}(t)}}(\mathbf {x}) > 0.1\}$, where $\mathbf {x}$ is the spatial coordinate. Instead, we use the notation $\Omega ^*$, $\Omega _{\mathrm{t}}^*$ and $\Omega _{\mathrm{h}}^*$ for the reference configurations of the whole brain, the tumour and the host tissue, respectively. Coherently, the tumour domain in the reference configuration is $\Omega _{\mathrm{t}}^* = \{ \mathbf {X} \in \mathbb {R}^3 \, : \, \chi _{{\Omega _{\mathrm{t}}^*}}(\mathbf {X}) > 0.1\}$, where $\mathbf {X}$ is the material coordinate. We remark that the tumour region $\Omega _{\mathrm{t}}^*$ in the reference configuration does not evolve in time. As mentioned above, brain tissue (both healthy and unhealthy) is regarded as a mixture of two phases: a solid one, with volume fraction $\phi _{\mathrm{s}}$, that represents the cellular component, and a liquid one, with volume fraction $\phi _\ell$, including the interstitial fluid  of the brain. The solid and fluid phases are considered to saturate all the available space, so that the condition $\phi _{\mathrm{s}} + \phi _\ell = 1$ holds at any point in the domain $\Omega (t)$ and at any time instant. Following standard definitions in mixtures theory, by knowing the true density ${\hat{\rho }}_{\alpha }$ of the material composing the $\alpha$-phase, with $\alpha \in \lbrace \mathrm s, \ell \rbrace$, it is possible to define the partial phase density $\rho _{\alpha }= {\hat{\rho }}_{\alpha } \phi _\alpha$. Then, we can introduce the displacement vector field $\mathbf {u}_{\mathrm{s}}$ of the solid phase, which defines the deformation of the body mapping the reference configuration to the current one, and the related deformation gradient $\mathbb {F}_{\mathrm{s}} = \mathbb {I} + \textrm{Grad} \, \mathbf {u}_{\mathrm{s}}$, with $\mathbb {I}$ being the second-order identity tensor and Grad denoting the gradient with respect to material coordinates.

**Fig. 1:**
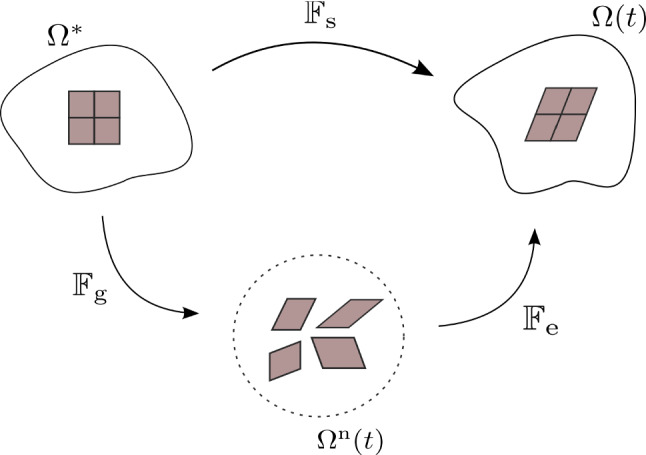
Schematics of the multiplicative decomposition of the deformation gradient

Furthermore, it is well known that a tissue undergoing growth, such as the one in the tumour region, experiences inelastic distortions and residual stresses (Skalak 1981; Rodriguez et al. 1994; Goriely 2017). To account for this fact from the mechanical point of view, a possible way is to employ a multiplicative decomposition of the deformation gradient (Ambrosi and Mollica 2002; Rajagopal 1995; DiCarlo and Quiligotti 2002): the tensor $\mathbb {F}_{\mathrm{s}}$ of the cellular population can therefore be split into two contributions, yielding

1 $${\mathbb{F}}_{{{\text{s}}}} = {\mathbb{F}}_{{{\text{e}}}} {\mathbb{F}}_{{{\text{g}}}}.$$

In Eq. (1), $\mathbb {F}_{\!\mathrm {e}}$ is the purely elastic contribution to the overall deformation gradient, whereas $\mathbb {F}_{\!\mathrm {g}}$ represents the inelastic distortions related to growth. The tensor $\mathbb {F}_{\!\mathrm {g}}$ determines the so-called *evolving natural state* $\Omega ^{\mathrm{n}}(t)$ of the body undergoing growth processes, where each material particle is allowed to grow freely and independently of the other ones. Hence, the natural state of the material is stress-free: the transition between the reference configuration and the natural state is then described by tensor $\mathbb {F}_{\mathrm{g}}$, while the subsequent elastic accommodation is included in $\mathbb {F}_{\mathrm{e}}$, because the state defined by $\mathbb {F}_{\mathrm{g}}$ is not in general compatible. We also recall that, throughout the path between the natural state and the current configuration, mass is assumed to be preserved, so that the growth contribution is entirely carried by $\mathbb {F}_{\mathrm{g}}$. A sketch of the multiplicative decomposition of the deformation gradient is reported in Fig. 1.

A consequence of Eq. (1) is that the volumetric part of the deformation gradient, $J_{\mathrm{s}} = \det \mathbb {F}_{\mathrm{s}}$, can be written as

2 $$\begin{aligned} J_{\mathrm{s}}=J_{\mathrm{e}} J_{\mathrm{g}}, \end{aligned}$$

with $J_{\mathrm{e}}:=\det \mathbb {F}_{\!\mathrm {e}}$ and $J_{\mathrm{g}}:=\det \mathbb {F}_{\!\mathrm {g}}$. Since the overall deformation gradient $\mathbb {F}_{\mathrm{s}}$ is assumed to be non-singular and $J_g \ge 1$, since we are considering growth processes, from Eq. (2) it follows that each tensor introduced in Eq. (1) is non-singular as well. Finally, we introduce the elastic right Cauchy–Green deformation tensor $\mathbb {C}_{\mathrm{e}} := \mathbb {F}_{\mathrm{e}}^{\mathrm{T}} \mathbb {F}_{\mathrm{e}}$ and its isochoric part $\overline{\mathbb {C}}_{\mathrm{e}} := J_{\mathrm{e}}^{-2/3} \mathbb {C}_{\mathrm{e}}$.

#### Mass and momentum balances

The multiphase approach we employ to describe tumour growth is based on the theory of mixtures and consists of a set of mass and momentum balance equations. First of all, we assume that the mixture is saturated and that both phases of the mixture have constant true densities, so that the materials composing the phases are incompressible. Then, since cells are mainly composed of water, we assume that the true densities of both phases are equal, i.e. ${\hat{\rho }}_{\mathrm{s}}={\hat{\rho }}_{{\ell }}$, and that external body forces (such as the gravitational force) as well as inertial effects are negligible: these hypotheses are reasonable when dealing with biological problems (Ambrosi and Preziosi 2002; Byrne and Preziosi 2003), since the motion of cells and interstitial fluid is very slow. Then, we write the balance equations for the cellular phase, with volume fraction $\phi _{\mathrm{s}}$ moving with velocity $\mathbf {v}_{\mathrm{s}}$, and the liquid phase, with volume fraction $\phi _\ell$ moving with velocity $\mathbf {v}_{\ell }$. As mentioned above, we remark that the boundary between the tumour and the healthy domain is advected with the velocity of the cell phase $\mathbf {v}_{\mathrm{s}}$. We assume that in the tumour region $\Omega _{\mathrm{t}}(t)$ cells proliferate, whereas in the domain occupied by the healthy tissue $\Omega _{\mathrm{h}}(t)$ the proliferation of cells is compensated by natural cell death, so that the net rate of growth $\Gamma _{\mathrm{s}}$ is equal to zero. Finally, assuming that the mixture is closed, the mass increase in the cellular phase happens at the expense of the liquid phase, so that the mass balances of the cellular and fluid phases read

3 $$\begin{aligned}&\frac{\partial \phi _{\mathrm{s}}}{\partial t} + \nabla \cdot (\phi _{\mathrm{s}} \mathbf {v}_{\mathrm{s}}) = \Gamma _{\mathrm{s}} \chi _{\Omega _{\mathrm{t}}(t)} \, , \end{aligned}$$

4 $$\begin{aligned}&\frac{\partial \phi _{\ell }}{\partial t} + \nabla \cdot (\phi _{\ell } \mathbf {v}_{\ell }) = \Gamma _{\ell } \chi _{\Omega _{\mathrm{t}}(t)} = - \Gamma _{\mathrm{s}} \chi _{\Omega _{\mathrm{t}}(t)} \, . \end{aligned}$$

The net rate of tumour growth $\Gamma _{\mathrm{s}}$ is influenced by many different factors, such as the availability of nutrients and the solid stress (Ambrosi and Mollica 2002, 2004; Ambrosi et al. 2017; Mascheroni et al. 2018). In a first approximation, one can assume that the amount of nutrients, denoted by its concentration $c_n$, along with the availability of space, are the main factors regulating cell growth, so that the following constitutive equation for the growth term holds

5 $$\begin{aligned} \Gamma _{\mathrm{s}}(\phi _{\mathrm{s}}, c_n) = {\gamma } \phi _{\mathrm{s}} (\phi _{\mathrm{max}} - \phi _{\mathrm{s}}) \left( c_n - c_0 \right) _{+}, \end{aligned}$$

where $( \cdot )_{+}$ denotes the positive part and $\gamma$ is a positive coefficient. That way, the proliferation rate depends affinely on the available concentration of nutrients $c_n$, provided that it is greater than a hypoxia threshold $c_0$. Conversely, when $c_n \le c_0$ the growth rate becomes zero and tumour expansion arrests. Moreover, in Eq. (5) we have that growth depends on the fraction of cells that is already present - which is reasonable since cell population grows by duplication; finally, we have a factor $(\phi _{\mathrm{max}} - \phi _{\mathrm{s}})$, whose presence is explained by the necessity to decrease the proliferation rate as the cellular phase fills all the available space for the solid constituent: this accounts for the phenomenon of contact inhibition of growth. More complex relations for $\Gamma _{\mathrm{s}}$ including explicitly the role of stresses may also be considered (Mascheroni et al. 2018, 2016; Stylianopoulos et al. 2013). Indeed, several studies have dealt with the effect of mechanical stresses on tumour growth in vitro, by embedding tumour spheroids either in agarose matrices of varying concentrations or in a culture medium with biocompatible polymers able to exert a mechanical stress, such as Dextran (Cheng et al. 2009; Helmlinger et al. 1997; Montel et al. 2012; Delarue et al. 2014). These studies showed that tumour growth is impaired by the compressive forces generated by the resistance of the surrounding tissue/matrix and that the mechanical stress has a strong impact on cancer progression. In the light of these observations, in the following we will also take into account an expression for the tumour proliferation rate that involves growth inhibition due to compressive stresses (Mascheroni et al. 2018), namely,

6 $$\begin{aligned} \Gamma _{\mathrm{s}}(\phi _{\mathrm{s}}, c_n, \Sigma ) = {\gamma } \phi _{\mathrm{s}} (\phi _{\mathrm{max}} - \phi _{\mathrm{s}}) \left( c_n - c_0 \right) _{+} \left( 1-\frac{\delta _1 \Sigma _+}{\Sigma _+ + \delta _2}\right) \, , \end{aligned}$$

where $\Sigma := -{\mathrm{tr}}(\mathbb {T}_{\mathrm{s}})/3$ is a measure of compression, related to the spherical part of the constitutive Cauchy stress tensor of the solid phase. The presence of the positive part means that, if the tissue is in compression, then growth is slowed down, while traction does not inhibit tumour proliferation. The constitutive definition of the stress $\mathbb {T}_{\mathrm{s}}$ as a function of the deformation will be provided in the next Subsection. Instead, $\delta _1$ and $\delta _2$ are parameters quantifying the inhibition of growth: in particular, $\delta _1 < 1$ regulates the maximum amount of inhibition due to stress, while $\delta _2$ describes how fast the reduction of $\Gamma _{\mathrm{s}}$ happens in response to compressive stresses.

In order to insert in the model the growth terms (5) and (6), it is necessary to introduce an equation describing the evolution of the nutrients in the domain. We assume that these chemicals are transported by the fluid phase and can diffuse into it; at the same time, they are taken by the growing tumour and uniformly supplied by the vasculature. We introduce the hypothesis that the nutrients uptake by the healthy tissue is negligible compared to the one by the tumour tissue: biologically, this is equivalent to saying that the nutrients absorbed by the host tissue are immediately replaced by the vasculature. Hence, if we denote by $c_n$ the concentration of available nutrients normalized with respect to the physiological concentration, so that $c_n \in [0,1]$, the mass balance of nutrients in $\Omega (t)$ reads

7 $$\begin{aligned} \dfrac{\partial }{\partial t} (\phi _{\ell } c_n) + \nabla \cdot (\phi _{\ell } c_n \mathbf {v}_{\ell }) = \nabla \cdot ( \phi _{\ell } \mathbb {D} \nabla c_n) + \Gamma _{\ell } c_{n} {\chi _{\Omega _{\mathrm{t}}(t)} }+ G_{n}\chi _{\Omega _{\mathrm{t}}(t)} \, , \end{aligned}$$

where $\mathbb {D}$ is the diffusion tensor, the term $\Gamma _{\ell } c_{n}$ accounts for the variation of the chemical concentration due to absorption/production of the liquid in which the chemical is dissolved, and $G_n$ models the supply of chemicals due to the presence of the blood vasculature and the consumption of nutrients by the cells in the tissue occurring without net variation of the liquid amount. This last term could represent the transport of nutrients/oxygen through the walls of the capillaries either without exchange of fluids, or with the possible excess of fluid due to the presence of leaky vessels in the tumour region automatically balanced by the venous capillaries and the lymphatic system (not explicitly included in the model). Thus, the exchanged fluid does not contribute to the growth/absorption of the constituent and the system remains closed with respect to the fluid and solid phases (Astanin and Preziosi 2008). Then, $G_n$ is multiplied by the indicator function of the tumour region, since this term is null in the healthy tissue where the nutrient supply is perfectly balanced in the physiological condition, whereas in the cancer region we have a higher consumption of nutrients due to the pathological proliferation of cells. The use of a tensor in the diffusion term allows to account for the structural anisotropy of brain tissue (Jbabdi et al. 2005), that induces fluids to diffuse preferentially along certain directions. Actually, the tensor $\mathbb {D}$, that can be obtained through DTI imaging and subsequent modification (see Sect. 2.2), describes how water diffuses along specific directions: however, if we consider that the main nutrient for cells is oxygen which is carried by water molecules, we can take the same tensor as a descriptor of the diffusion values of nutrients. For what concerns the nutrients source term, in this work we will consider the following form

8 $$\begin{aligned} G_{n}(\phi _{\mathrm{s}}, \phi _\ell , c_n) = -\zeta \phi _{\mathrm{s}} \phi _\ell c_n + S_n (1-c_n) \phi _\ell \, . \end{aligned}$$

This expression describes the fact that nutrients are consumed by the tumour with a constant rate $\zeta$: the uptake depends on the volumetric fractions of cells and liquid in the tumour region, as well as on the available concentration of nutrients. Concurrently, nutrients are supplied by the vasculature at a constant rate $S_n$ as long as their concentration is below the physiological value, i.e. $c_n < 1$, and they are dispersed in the liquid phase. The whole expression is multiplied by the tumour indicator function in Eq. (7), since, as mentioned above, in the healthy region we assume that production and absorption of nutrients are reciprocally balanced. By using standard calculus techniques and recalling the mass balance equation of the fluid phase (4) and the functional formulation of $G_n$ assumed in (8), Eq. (7) can be rephrased as

9 $$\begin{aligned} \frac{\partial c_n}{\partial t} + \mathbf {v}_{\ell } \cdot \nabla c_n = \dfrac{1}{\phi _\ell } \nabla \cdot ( \phi _{\ell } \mathbb {D} \nabla c_n) + \left[ - \zeta \phi _{\mathrm{s}} c_n + S_n (1 - c_n) \right] \chi _{\Omega _{\mathrm{t}}(t)}. \end{aligned}$$

As regards the momentum balances, we recall that, in a saturated mixture, the partial Cauchy stress tensor associated with the $\alpha$-th phase of the mixture can be written as

10 $$\begin{aligned} {\widetilde{\mathbb {T}}}_{\alpha } = - \phi _{\alpha } \,p \mathbb {I} + \mathbb {T}_{\alpha }, \end{aligned}$$

where $\mathbb {T}_{\alpha }$ is referred to as *effective* (or extra-) stress, and the purely hydrostatic contribution $- \phi _{\alpha } \, p \mathbb {I}$ indicates the amount of pressure sustained by the $\alpha$-th phase. We underline that, in the present theory, *p* is a Lagrange multiplier related to the mixture incompressibility, rather than a constitutively determined quantity. Moreover, in the following we will neglect both the inertial effects and the momentum exchange rates between phases associated with the mass sources/sinks $\Gamma _\alpha$, $\alpha \in \{ {\mathrm{s}}, \ell \}$. These assumptions are reasonable in the context of biological growth, which is a process that takes place on long time scales with small velocities for both the phases of the mixture (Giverso et al. 2015). Then, taking into account these observations, the momentum balance for each phase reads

11 $$\begin{aligned}&-p \nabla \phi _{\mathrm{s}} - \phi _{\mathrm{s}} \nabla p + \nabla \cdot \mathbb {T}_{\mathrm{s}} + \widetilde{\mathbf {m}}_{{\mathrm{s}}\ell } = \mathbf {0}, \end{aligned}$$

12 $$\begin{aligned}&-p \nabla \phi _\ell - \phi _{\ell } \nabla p + \nabla \cdot \mathbb {T}_{\ell } + \widetilde{\mathbf {m}}_{\ell {\mathrm{s}}} = \mathbf {0}, \end{aligned}$$

where the term $\widetilde{\mathbf {m}}_{\alpha \beta }$ represents the force acting on the $\alpha$-th phase due to the other phase $\beta$. In particular, following thermodynamical prescriptions, the latter can be decomposed as $\widetilde{\mathbf {m}}_{\alpha \beta }~=~p \nabla \phi _\alpha + \overline{\mathbf {m}}_{\alpha \beta }$, highlighting the non-dissipative and dissipative contributions, respectively (Giverso et al. 2015; Hassanizadeh 1986). Coherently with the hypotheses usually made to deduce Darcy’s law, we require that the extra-stress of the fluid phase $\mathbb {T}_{\ell }$ is negligible with respect to the pressure gradient and to the dissipative interaction forces between fluid and solid phase, that can be assumed in the form $\overline{\mathbf {m}}_{\ell {\mathrm{s}}} = - \mu \phi _{\ell }^2\mathbb {K}^{-1}(\phi _{\ell }) (\mathbf {v}_{\ell } - \mathbf {v}_{\mathrm{s}})$ (Giverso et al. 2015), where $\mathbb {K}$ is the permeability tensor and μ is the dynamic viscosity of the fluid component. As a consequence, from Eq. (12) the classical Darcy’s law as a momentum balance for the fluid phase is retrieved

13 $$\begin{aligned} \mathbf {v}_{\ell } = \mathbf {v}_{\mathrm{s}} - \frac{\mathbb {K}(\phi _{\ell })}{\mu \phi _{\ell }} \nabla p . \end{aligned}$$

Then, the momentum balance for the mixture as a whole can be obtained by summing Eqs. (11)–(12), taking into account the saturation condition $\phi _{\mathrm{s}} + \phi _\ell = 1$ and the action–reaction principle $\overline{\mathbf {m}}_{{\mathrm{s}}\ell } = - \overline{\mathbf {m}}_{\ell {\mathrm{s}}}$

14 $$\begin{aligned} - \nabla p + \nabla \cdot \mathbb {T}_{\mathrm{s}} = \mathbf {0}. \end{aligned}$$

We remark that the action–reaction condition applies in principle to the interaction forces between phases, i.e. $\widetilde{\mathbf {m}}_{\mathrm{s}\ell} + \widetilde{\mathbf {m}}_{\ell {\mathrm{s}}}= \mathbf {0}$. However, since the non-dissipative contributions to $\widetilde{\mathbf {m}}_{\mathrm{s}\ell}$ and $\widetilde{\mathbf {m}}_{\ell \mathrm s}$ are given by $p\nabla \phi _{\mathrm{s}}$ and $p \nabla \phi _\ell$, respectively, it follows from the saturation condition that the constraint also holds for the dissipative parts, leading to $\overline{\mathbf {m}}_{{\mathrm{s}}\ell } + \overline{\mathbf {m}}_{\ell {\mathrm{s}}} = \mathbf {0}$.

To model the presence of white and grey matter fibres in the brain tissue and account for the consequent anisotropy in fluid motion, we will take the permeability tensor as

15 $$\begin{aligned} \mathbb {K}(\phi _{\ell }) = \mu \hat{k}(\phi _\ell ) \mathbb {A}, \end{aligned}$$

where $\mathbb {A}$ denotes the *tensor of preferential directions* (Colombo et al. 2015) derived through DTI imaging, whose construction will be described in Sects. 2.2 and 3. This approach allows to model preferential fluid and nutrients motion along the fibre tracts in the brain, taking into account the anisotropic structure of the tissue. Instead, the coefficient $\hat{k}(\phi _\ell )$ is given by the exponential Holmes–Mow expression (Holmes and Mow 1990; Mow et al. 1980), as it is often done for soft tissues (Guo et al. 2014; Di Stefano et al. 2019)

16 $$\begin{aligned} \hat{k}(\phi _\ell ) = k(J_{\mathrm{e}}) = k_0 \left( \frac{J_{\mathrm{e}}-\phi _{\mathrm{sn}}}{1-\phi _{\mathrm{sn}}}\right) ^{\alpha _0} e^{m(J_{\mathrm{e}}^2-1)/2}, \end{aligned}$$

where $\alpha _0$ and *m* are model parameters, $k_0$ is a reference value for *k* taken in the natural state, and $\phi _{\mathrm{sn}}$ is the volume fraction of the solid phase in the natural state. An estimate of all parameters will be provided in Sect. 2.2.

#### Constitutive equations for the stress tensors

To close the system of mass and momentum balance equations, derived in the previous Subsection, it is necessary to determine an appropriate evolution law for the Cauchy stress tensor $\mathbb {T}_{\mathrm{s}}$ associated with the cellular population, both in the diseased and in the healthy region, i.e.

17 $$\begin{aligned} \mathbb {T}_{\mathrm{s}} = \mathbb {T}_{\mathrm{s}}^{\mathrm{t}} \, \chi _{\Omega _{\mathrm{t}}(t)} + \mathbb {T}_{\mathrm{s}}^{\mathrm{h}} \left(1-\chi _{\Omega _{\mathrm{t}}(t)}\right) \qquad \text { in }\quad \Omega (t)\, , \end{aligned}$$

where $\mathbb {T}_{\mathrm{s}}^{\mathrm{t}}$ is the Cauchy stress tensor associated with the tumour cells and $\mathbb {T}_{\mathrm{s}}^{\mathrm{h}}$ is the Cauchy stress tensor associated with the healthy cells. This is a relevant part of the mathematical model, since our primary aim is to study how brain tumour growth influences mechanically the surrounding tissues and to quantify the entity of stress and deformation as a consequence of abnormal proliferation. We remark that several difficulties arise when dealing with experimental settings involving brain tissue and the definition of a realistic constitutive equation is a non-trivial problem that is still debated (Budday et al. 2020; Chatelin et al. 2010). Most of the brain biomechanical studies performed in the last fifty years have been done in vitro on excised samples of brains (either from humans, when available, or from animals) with different experimental protocols, that make the results difficult to be compared. Moreover, in vitro tests need to be generalized to in vivo conditions, providing additional complications. However, novel techniques and protocols have been recently proposed in the literature to carry out in vivo non-destructive and non-invasive investigations. In particular, magnetic resonance elastography (MRE) emerged as the most promising non-invasive imaging technique to measure the mechanical parameters of biological soft tissues by coupling a mechanical excitation, which promotes elastic wave propagation in the soft medium, to a magnetic resonance imaging (MRI) device for visualizing it (Chatelin et al. 2010). Several classes of algorithms exist for quantitatively estimating the stiffness from the analysis of wave propagation: the most commonly used are based on time points sampling designed to solve the inversion problems under the assumption of linear elasticity (Murphy et al. 2019; Fehlner et al. 2017). The identification of mechanical parameters from MRE using nonlinear elasticity is still under investigation. Furthermore, the mechanical properties obtained from MRE are sensitive to positioning, to the method for extracting elastic parameters, and to the excitation frequency. Therefore, nowadays, the use of MRE does not offer enough information to establish nonlinear, finite strain constitutive models for realistic computational simulations (Budday et al. 2020) and the use of in vitro experiments to characterize the brain and tumour elastic properties in a nonlinear regime (such as the one occurring during tumour growth) is still the most established. In the context of *in vitro* experiments, a first important issue put forward by experimental studies (Goriely et al. 2015; Budday et al. 2017; de Rooij and Kuhl 2016) concerns the anisotropy of brain tissue: despite the intrinsic microstructural anisotropy due to the presence of nerve tracts, the human brain tissue seems nearly isotropic from a mechanical viewpoint and no significant directional dependency affecting the mechanical behaviour can be observed, even in highly anisotropic regions of the brain. Therefore, the brain can be considered isotropic as far as mechanics is concerned, whereas anisotropy cannot be neglected when dealing with the diffusion of substances and with fluids and cell motion (Budday et al. 2017). As regards the constitutive characterization, the vast majority of experimental results agree upon the highly nonlinear and viscoelastic nature of brain tissue (Goriely et al. 2015; Budday et al. 2017; de Rooij and Kuhl 2016), under different loading conditions (Rashid et al. 2012, 2014, 2013; Miller et al. 2000) and even with multiple loading modes (Budday et al. 2017). However, for the purposes of our work, we are interested in brain response under small strain rates induced by cell proliferation: therefore, the rate dependent response can be neglected without introducing significant errors (Ambrosi and Mollica 2002). To describe the elastic response, several models have been proposed in the literature (de Rooij and Kuhl 2016) and there is a common agreement that the generalized Ogden model (Ogden 1972) is suitable to represent the mechanical behaviour of soft brain tissue. In particular, the Mooney–Rivlin model, which is a particular case of the generalized Ogden energy, turns out to be an appropriate choice from the experimental point of view (Mihai et al. 2017; Balbi et al. 2019; Destrade et al. 2015). We further remark that the fitting of the experimental data to get a quantitative estimation of the behaviour of the brain is generally obtained under the assumption of incompressibility of the sample described as a solid, without taking into account the contribution of the liquid encapsulated inside it. However, brain tissues have an exceptionally high water content in vivo (Budday et al. 2020) and are better represented by a mixture of at least two constituents, a liquid and a solid phase. As stated before, the constituents composing the mixture are said to be incompressible if their true densities ${\hat{\rho }}^{\alpha }$ are constants. The bulk density $\rho ^{\alpha }$ needs not be constant even if the $\alpha$-constituent is incompressible and, thus, $J_{\mathrm{e}}$ is not constrained to be equal to 1. The variation of $J_{\mathrm{e}}$ leads to a deformation of the pores, that in turn induces volumetric solid stresses. To take into account the experimental observations on the mechanical behaviour of the brain under isochoric conditions and the existence of a volumetric stress due to variations of $J_{\mathrm{e}}$, the strain energy density function for both the solid tumour mass $\mathcal {W}_{\mathrm{sn}}^{\,\mathrm{t}}$ and the healthy brain tissue $\mathcal {W}_{\mathrm{sn}}^{\,\mathrm{h}}$, expressed per unit volume of the natural state $\Omega ^{\mathrm{n}}(t)$, is additively split into an isochoric part, $\widehat{\mathcal {W}}_{\mathrm{sn}}^{\,\omega \mathrm {i}}$, and a volumetric part, $\widehat{\mathcal {W}}_{\mathrm{sn}}^{\,\omega \mathrm {v}}$:

18 $$\begin{aligned} {\mathcal {W}_{\mathrm{sn}}^{\,\omega }(\mathbb {C}_{\mathrm{e}}) = \widehat{\mathcal {W}}_{\mathrm{sn}}^{\,\omega }(\overline{\mathbb {C}}_{\mathrm{e}}, J_{\mathrm{e}}) = \widehat{\mathcal {W}}_{\mathrm{sn}}^{\,\omega \mathrm i}(\overline{\mathbb {C}}_{\mathrm{e}}) + \widehat{\mathcal {W}}_{\mathrm{sn}}^{\,\omega \mathrm v}(J_{\mathrm{e}}) \, , } \end{aligned}$$

with $\omega \in \lbrace \mathrm {t}, \mathrm {h} \rbrace$. We remark that many of the strain energy density functions used to represent brain tissues, such as the Mooney–Rivlin model used hereafter, can be naturally written in the separable form of Eq. (18). Furthermore, even in those cases in which the contribution related to $J_{\mathrm{e}}$ cannot be decoupled from the one related to $\overline{\mathbb {C}}_{\mathrm{e}}$, for small variations of $J_{\mathrm{e}}$ (i.e. in the case of approximately elastically incompressible materials (Gurtin et al. 2010)), it is always possible to approximate the strain energy density function with such a separable form. Then, even though, in principle, the mechanical model for the tumour tissue might be taken as totally different from the one describing the elastic behaviour of the healthy tissue (Stewart et al. 2017), in the following we assume the same functional form for the strain energy density functions both in the tumour and in the healthy region, with possibly varying mechanical parameters. Specifically, following (Balbi et al. 2019; Destrade et al. 2015), we take a Mooney–Rivlin model for the isochoric strain energy density function, i.e. for $\omega \in \lbrace \mathrm {t}, \mathrm {h} \rbrace$

19 $$\begin{aligned} \widehat{\mathcal {W}}_{\mathrm{sn}}^{\omega \mathrm {i}}(\overline{\mathbb {C}}_{\mathrm{e}})&= \dfrac{1}{2}\mu _{1}^{\omega } \left( \mathrm {I}_{\overline{\mathbb {C}}_{\mathrm{e}}} - 3 \right) + \dfrac{1}{2}\mu _{2}^{\omega }\left( \mathrm {II}_{\overline{\mathbb {C}}_{\mathrm{e}}} - 3 \right) \, , \end{aligned}$$

where $\mathrm {I}_{\overline{\mathbb {C}}_{\mathrm{e}}} = {{\,\mathrm{tr}\,}}(\overline{\mathbb {C}}_{\mathrm{e}})$, $\mathrm {II}_{\overline{\mathbb {C}}_{\mathrm{e}}} =\frac{1}{2} \left[ \left( {{\,\mathrm{tr}\,}}\, \overline{\mathbb {C}}_{\mathrm{e}} \right) ^2 - {{\,\mathrm{tr}\,}}\left( \overline{\mathbb {C}}_{\mathrm{e}}^2 \right) \right]$ are, respectively, the first and second principal invariant of $\overline{\mathbb {C}}_{\mathrm{e}}$. The material parameters of the cancer tissue, $\mu _{1}^{\mathrm{t}}$ and $\mu _{2}^{\mathrm{t}}$, are in general different from the ones employed to describe the healthy brain tissue, $\mu _{1}^{\mathrm{h}}$ and $\mu _{2}^{\mathrm{h}}$. For what concerns the volumetric part $\widehat{\mathcal {W}}_{\mathrm{sn}}^{\,\omega \mathrm {v}}$, with $\omega \in \lbrace \mathrm {t}, \mathrm {h} \rbrace$, we take the following form (Gurtin et al. 2010; Horgan and Saccomandi 2004; Prevost et al. 2011):

20 $$\begin{aligned} {\widehat{\mathcal {W}}_{\mathrm{sn}}^{\,\omega \mathrm {v}}(J_{\mathrm{e}}) = \dfrac{1}{2}\kappa ^{\mathrm{\omega }} \, (\ln J_{\mathrm{e}})^2 \, ,} \end{aligned}$$

where $\kappa ^{\omega }$ is the elastic parameter associated with the response of the tumour and healthy tissue to volumetric deformations. Other functional forms for $\widehat{\mathcal {W}}_{\mathrm{sn}}^{\omega \mathrm {v}}(J_{\mathrm{e}})$ taking into account the concept of the compaction point (Ehlers and Eipper 1999) or the existence of a maximum cell volume fraction (Byrne and Preziosi 2003) could alternatively be used. Once a proper constitutive form for the strain energy density function $\mathcal {W}_{\mathrm{sn}}^{\,\omega }$ is chosen, it is possible to compute the constitutive part of the solid phase stress tensor inside the tumour and the healthy regions:

21 $$\begin{aligned} \mathbb {T}_{\mathrm{s}}^{\omega }&= 2 J_{\mathrm{e}}^{-1} \mathbb {F}_{\mathrm{e}} \frac{\partial \widehat{\mathcal {W}}_{\mathrm{sn}}^{\omega }(\overline{\mathbb {C}}_{\mathrm{e}}, J_\mathrm{e})}{\partial \mathbb {C}_{\mathrm{e}}} \mathbb {F}_{\mathrm{e}}^{\mathrm{T}} = { J_{\mathrm{e}}^{-1} \mathbb {F}_{\mathrm{e}} \mathbb {S}_{\mathrm{sn}}^{\omega } \mathbb {F}_{\mathrm{e}}^{\mathrm{T}}\,} , \end{aligned}$$

where $\mathbb {S}_{\mathrm{sn}}^{\omega }$ is the solid phase second Piola–Kirchhoff stress tensor associated with the natural state, in the $\omega \in \lbrace \mathrm {t}, \mathrm {h} \rbrace$ domain.

By classical computations (Gurtin et al. 2010) one obtains

22 $$\begin{aligned} {\mathbb {S}_{\mathrm{sn}}^{\,\omega}}&{= 2 \frac{\partial \widehat{\mathcal {W}}_{\mathrm{sn}}^{\omega }(\overline{\mathbb {C}}_{\mathrm{e}}, J_\mathrm{e})}{\partial \mathbb {C}_{\mathrm{e}}}} \nonumber \\&{ = 2 J_{\mathrm{e}}^{-2/3} \left( \underline{\underline{\mathbb {I}}} -\frac{1}{3}\overline{\mathbb {C}}_\mathrm{e}^{-1} \otimes \overline{\mathbb {C}}_{\mathrm{e}}\right) : \frac{\partial \widehat{\mathcal {W}}_{\mathrm{sn}}^{\,\omega}(\overline{\mathbb {C}}_{\mathrm{e}}, J_\mathrm{e})}{\partial \overline{\mathbb {C}}_{\mathrm{e}}} + J_{\mathrm{e}}\frac{\partial \widehat{\mathcal {W}}_{\mathrm{sn}}^{\,\omega}(\overline{\mathbb {C}}_{\mathrm{e}}, J_\mathrm{e})}{\partial J_{\mathrm{e}}}\mathbb {C}_{\mathrm{e}}^{-1}} \nonumber \\&{ = 2 J_{\mathrm{e}}^{-2/3} \left( \underline{\underline{\mathbb {I}}} -\frac{1}{3}\overline{\mathbb {C}}_{\mathrm{e}}^{-1} \otimes \overline{\mathbb {C}}_{\mathrm{e}}\right) : \frac{\partial \widehat{\mathcal {W}}_{\mathrm{sn}}^{\,\omega \mathrm i}(\overline{\mathbb {C}}_{\mathrm{e}})}{\partial \overline{\mathbb {C}}_{\mathrm{e}}} + J_{\mathrm{e}}\frac{\partial \widehat{\mathcal {W}}_{\mathrm{sn}}^{\,\omega \mathrm v}(J_\mathrm{e})}{\partial J_{\mathrm{e}}}\mathbb {C}_{\mathrm{e}}^{-1} } \, , \end{aligned}$$

where $\underline{\underline{\mathbb {I}}}$ is the symmetric fourth-order identity tensor, with components $\underline{\underline{\mathbb {I}}}_{\,ijkl}~=~\frac{1}{2}\left(\delta _{ik}\delta _{jl} + \delta _{il}\delta _{jk}\right)$, and the tensor product $\mathbb {A}\otimes \mathbb {B}$ of two second-order tensors is defined by $(\mathbb {A}\otimes \mathbb {B}):\mathbb {H}=(\mathbb {B}:\mathbb {H})\mathbb {A}$. From (22) it is possible to define the fourth-order elasticity tensor of the solid phase

$$\underline{{\underline{\mathbb{C}} }}_{\,\textrm{sn}}^{\omega } = 2\frac{{\partial {\mathbb{S}}_{\textrm{sn}}^{\omega} }}{{\partial \mathbb{C}_{\textrm{e}} }} \, ,\qquad \omega \in \{ t,h\} \, .$$

As a consequence of Eqs. (18) and (21), the Cauchy stress tensor of the solid phase is decomposed into a deviatoric part $\mathbb {T}_{\mathrm{s}}^{\omega \mathrm d}$, for which we have ${{\,\mathrm{tr}\,}}(\mathbb {T}_{\mathrm{s}}^{\omega \mathrm d}) = 0$, and a spherical component $\mathbb {T}_{\mathrm{s}}^{\omega \mathrm v}$, with $\omega \in \{{\mathrm{t}}, {\mathrm{h}}\}$, i.e.

23 $$\begin{aligned} { \mathbb {T}_{\mathrm{s}}^{\omega } }&{ = 2 J_{\mathrm{e}}^{-1} \left[ J_{\mathrm{e}}^{-2/3} \mathbb {F}_{\mathrm{e}} \frac{\partial \widehat{\mathcal {W}}_{\mathrm{sn}}^{\,\omega \mathrm i}(\overline{\mathbb {C}}_{\mathrm{e}})}{\partial \overline{\mathbb {C}}_{\mathrm{e}}} \mathbb {F}_{\mathrm{e}}^{\mathrm{T}} - \frac{1}{3} \left( \overline{\mathbb {C}}_{\mathrm{e}} : \frac{\partial \widehat{\mathcal {W}}_{\mathrm{sn}}^{\,\mathrm{\omega i}}(\overline{\mathbb {C}}_{\mathrm{e}})}{\partial \overline{\mathbb {C}}_{\mathrm{e}}}\right) \mathbb {I} \right] + \frac{\partial \widehat{\mathcal {W}}_{\mathrm{sn}}^{\,\omega \mathrm v}(J_{\mathrm{e}})}{\partial J_{\mathrm{e}}} \mathbb {I} } \nonumber \\&{= \mathbb {T}_{\mathrm{s}}^{\omega \mathrm d} + \mathbb {T}_{\mathrm{s}}^{\omega \mathrm v} \, . } \end{aligned}$$

The constitutive expressions of the Cauchy stress tensors $\mathbb {T}_{\mathrm{s}}^{\mathrm{t}}$ and $\mathbb {T}_{\mathrm{s}}^{\mathrm{h}}$ must be accompanied by equations defining $\mathbb {F}_{\!\mathrm {s}}$ and $\mathbb {F}_{\!\mathrm {g}}$. However, the deformation gradient tensor $\mathbb {F}_{\!\mathrm {s}}$, which is entirely determined by the motion of the cell phase, is not an additional unknown for the model, whereas $\mathbb {F}_{\!\mathrm {g}}$ has to be determined by solving appropriate evolution equations. The evolution of $\mathbb {F}_{\!\mathrm {g}}$ can be obtained self-consistently by working out Eq. (3) (Giverso et al. 2015; Mascheroni et al. 2018; Grillo et al. 2012). In particular, we assume that the orientation of the mitotic spindle of cell division, which could be affected by external mechanical cues and by the mechanical behaviour of the tissue (here taken as isotropic), is not influenced by brain fibre alignment, so that growth deformations are isotropic as well. Therefore, the inelastic tensor can be written as

24 $${\mathbb{F}}_{{{\text{g}}}} = {\text{g}}{\mathbb{I}},$$

with *g* a scalar field whose evolution is given by an ordinary differential equation (Ambrosi and Mollica 2002; Grillo et al. 2012):

25 $$\frac{{\dot{g}}}{g} = \frac{1}{3}\frac{{\Gamma _{{\text{s}}} }}{{\phi _{{\text{s}}} }}\chi _{{\Omega _{{\text{t}}}^{*} }} \qquad {\text{ in }}\quad\Omega ^{*} .$$

We remark that, even if the multiplicative decomposition of the deformation gradient is actually needed only inside the tumour region, for simplicity we assume its validity everywhere in the domain. Thus, as a consequence of Eqs. (24)–(25), we will have $\mathbb {F}_{\mathrm{g}} = \mathbb {I}$ outside the cancer domain.

### Parameters estimation

A fundamental passage to complete the mathematical model and focus on its numerical implementation consists in assessing the values of the parameters that appear in the system. This is both a challenging and delicate task: since our goal is to simulate tumour progression and its mechanical impact, the choice of the parameters is crucial to have a realistic and reliable outcome. At the same time, when working in the field of mathematical biology, accurate estimations of the parameters are often difficult to obtain: this is particularly true for the brain, which is very difficult to be investigated experimentally (Goriely et al. 2015). In this Section, we review the literature so as to assign a value, or at least a range of values, to the parameters introduced in our model, in order to test its qualitative behaviour.

First of all, we deal with the material parameters $\mu _1^{\mathrm{t}}$, $\mu _2^{\mathrm{t}}$, $\mu _1^{\mathrm{h}}$, $\mu _2^{\mathrm{h}}$ that appear in the Mooney–Rivlin energy densities. We take as a reference the work by Balbi et al. (2019), who analysed the constitutive behaviour of brain matter considering a Mooney–Rivlin-type energy, for which they propose as mean values for the material parameters $\mu _1^{\mathrm{h}} = 153$ Pa and $\mu _2^{\mathrm{h}} = 297$ Pa. We consider these values as references for the healthy tissue mechanics. As regards tumour tissue, several experimental studies have assessed that it is in general stiffer than the healthy one: Stewart et al. (2017) showed that human brain tumours like gliomas and meningiomas are two to five times stiffer than normal brain tissue; Chauvet et al. (2016) and Miroshnikova et al. (2016) proved a significant increase in stiffness for high-grade gliomas, more than ten times the healthy reference value (Clatz et al. 2005; Agosti et al. 2020). Therefore, for our main simulations we take the material parameters in the tumour region as ten times greater than the ones in the healthy region. However, some works estimate the stiffness of brain tumours to be either the same order as the healthy tissue or even softer (Nia et al. 2017; Svensson et al. 2022). Hence, to compare the growth velocities, we will also consider a case in which the parameters differ by four times and a case in which the tumour and the host tissue are assigned the same mechanical parameters, equal to the ones of the normal brain. For what concerns the volumetric moduli $\kappa ^{\mathrm{t}}$ and $\kappa ^{\mathrm{h}}$, as mentioned above they penalize volumetric changes in the solid skeleton. However, their estimation is difficult, since most of the experimental works and subsequent modelling do not consider the brain as a mixture. In some previous works on biological tissues considered as porous media, the coefficient related to the volume excess stress is evaluated using the Young modulus (Byrne and Preziosi 2003; Agosti et al. 2018a; Colombo et al. 2015), which is very low for the brain (Clatz et al. 2005; Agosti et al. 2020; Budday et al. 2020). The work by Prevost et al. (2011), using a volumetric brain tissue response comparable to the one used in the present work, estimates a range of $2\times 10^{2} - 2\times 10^{4}$ Pa for the volumetric modulus. Therefore, following these observations and taking into account that the brain is very soft, we choose $\kappa ^{\mathrm{t}} = 1.4 \times 10^{-3}$ MPa and $\kappa ^{\mathrm{h}} = 1.4 \times 10^{-4}$ MPa, looking forward to further experimental confirmation.

As regards the parameters involved in the growth rate $\Gamma _\mathrm{s}$ proposed in Eq. (5), we estimate them as done in other recent works on brain tumours (Colombo et al. 2015; Agosti et al. 2018a). In particular, the cell proliferation constant $\gamma$ is taken as the inverse of typical doubling times for in vitro glioma cells, that vary from 24 to 48 hours: then, a range $0.5 - 1$ $\hbox {day}^{-1}$ can be considered appropriate for $\gamma$ (Frieboes et al. 2007). Since proliferation depends significantly on nutrients availability, also smaller values seem however admissible (Colombo et al. 2015): for this reason, in the following we will consider the minimum value inside the mentioned interval, i.e. $\gamma = 0.5$ $\hbox {day}^{-1}$. The hypoxia threshold $c_0$ is estimated in the literature as ranging from 0.15 to 0.5 (Gerlee and Anderson 2007; Frieboes et al. 2007; Tanaka et al. 2009): we will consider an intermediate value of $c_0 = 0.3$ in simulations. Moreover, we need to estimate the nutrients consumption rate $\zeta$ and the nutrients supply rate $S_n$ appearing in Eq. (8): as far as the former is concerned, following the approach by Colombo et al. (2015), it can be estimated indirectly through biological measurements of the oxygen diffusion coefficient in the human brain $D_n$ and the distance covered by an oxygen molecule before it is uptaken by a cancer cell $l_n$. The mean value for $D_n$ reported in the literature is $D_n = 86.4$ $\hbox {mm}^2/\text{day}$ (Colombo et al. 2015; Frieboes et al. 2007), while $l_n$ is estimated to be about $l_n = 10^{-1}$ mm (Frieboes et al. 2007). Hence, we can take a value of $\zeta = D_n / l_n^2 = 8640$ $\hbox {days}^{-1}$. The parameter $S_n$ is instead quite difficult to estimate: as done in Colombo et al. (2015), Agosti et al. (2018) we refer to the value of $10^{4}$ $\hbox {days}^{-1}$ proposed in Chatelain et al. (2011).

When we consider the stress-inhibited proliferation rate defined in Eq. (6), the parameters governing the impairment of growth due to compression $\delta _1$ and $\delta _2$ have to be estimated as well. Referring to Mascheroni et al. (2018), in our simulations we will consider $\delta _1 = 0.8 - 0.9$, while we will choose $\delta _2 = 10^{-3} -10^{-4}$ MPa to investigate different sensitivities to growth inhibition.

Regarding the estimate of $\phi _{\mathrm{sn}}$, that is, the cell volumetric fraction in the natural state, it is usually assumed to be a constant given from the outset (Ambrosi and Mollica 2002; Grillo et al. 2012). Different values appear in the literature: Colombo et al. (2015) and Agosti et al. (2018a) in their model for GBM considered a value of $\phi _{\mathrm{sn}} = 0.39$, which they derived as the complementary value of the extracellular space studied in Bruehlmeier et al. (2003) and amounting at up to $61 \%$. In our simulations, coherently with the constraint $\phi _{\mathrm{max}} = 0.85$ lower than 1, we set $\phi _{\mathrm{sn}} = 0.3$.

Finally, it remains to estimate the parameters which appear in the permeability tensor expression from Eq. (16), and in particular inside $\hat{k}(\phi _\ell )$. Given its definition and the spatial and temporal scales we employ in our model, this function has units $\hbox {mm}^2$/(MPa $\cdot$ day). As usually done for the Holmes–Mow permeability in soft tissues (Guo et al. 2014; Di Stefano et al. 2019), the values $\alpha _0 = 0.0848$ and $m = 4.638$ are considered. Concerning the reference permeability $k_0$, values found in the literature for the brain cover a range of $10^{4}-10^{5}$ $\hbox {mm}^2 /(\text{MPa} \cdot \text{day})$: for instance, Mascheroni et al. (2018) consider a value of $4.2 \times 10^{4}$ $\hbox {mm}^2$/(MPa $\cdot$ day) for the fluid phase in GBM tumour spheroids, modelled as mixtures. Instead, Basser (1992) proposed values of $4.31\times 10^{5} - 6.47 \times 10^5$ $\hbox {mm}^2$/(MPa $\cdot$ day) for the permeability of white and grey matter, respectively. Coherently, Smith and Humphrey (2007) reported a range of $1.47 \times 10^5 - 2.67 \times 10^5$ $\hbox {mm}^2$/(MPa $\cdot$ day), while a conversion of the value used by Jin et al. (2016) leads to $7.8 \times 10^4$ $\hbox {mm}^2$/(MPa $\cdot$ day). Finally, in Asgari et al. (2016) a value of $1.72 \times 10^5$ $\hbox {mm}^2$/(MPa $\cdot$ day) was employed. Therefore, we choose to consider an intermediate value of $k_0 = 2.17 \times 10^5$ $\hbox {mm}^2$/(MPa $\cdot$ day).

We report the complete list of parameters, along with their description, their values, and the main references, in Table 1.

**Table 1:** Values of model parameters

Parameter	Description	Value	Reference
$\mu _{1}^{\mathrm{{t}}}$	Mooney–Rivlin parameter (tumour)	1.53 $\times$ 10$^{-3}$ MPa	Chauvet et al. (2016); Miroshnikova et al. (2016)
$\mu _{2}^{\mathrm{{t}}}$	Mooney–Rivlin parameter (tumour)	2.97 $\times$ 10$^{-3}$ MPa	Chauvet et al. (2016); Miroshnikova et al. (2016)
$\kappa ^{\mathrm{t}}$	Volumetric modulus (tumour)	1.40 $\times$ 10$^{-3}$ MPa	Prevost et al. (2011)
$\mu _{1}^{\mathrm{{h}}}$	Mooney–Rivlin parameter (healthy)	1.53 $\times$ 10$^{-4}$ MPa	Balbi et al. (2019)
$\mu _{2}^{\mathrm{{h}}}$	Mooney–Rivlin parameter (healthy)	2.97 $\times$ 10$^{-4}$ MPa	Balbi et al. (2019)
$\kappa ^{\mathrm{h}}$	Volumetric modulus (healthy)	1.40 $\times$ 10$^{-4}$ MPa	Prevost et al. (2011)
$\gamma$	Cell proliferation constant	0.5 $\hbox {day}^{-1}$	Frieboes et al. (2007)
$c_0$	Hypoxia threshold	0.3	Gerlee and Anderson (2007)
$\zeta$	Nutrients consumption rate	8640 $\hbox {day}^{-1}$	Frieboes et al. (2007)
$S_n$	Nutrients supply rate	10$^{4}$ $\hbox {day}^{-1}$	Colombo et al. (2015)
$\delta _1$	Parameter related to growth inhibition	$0.8-0.9$	Mascheroni et al. (2018)
$\delta _2$	Parameter related to growth inhibition	$10^{-3}-10^{-4}$ MPa	Mascheroni et al. (2018)
$\phi _{\mathrm{sn}}$	Cell volume fraction (natural state)	0.3	Giverso et al. (2015)
$\phi _{\mathrm{max}}$	Maximum cell volume fraction	0.85	–
$\alpha_0$	Holmes–Mow permeability parameter	0.0848	Guo et al. (2014); Di Stefano et al. (2019)
*m*	Holmes–Mow permeability parameter	4.638	Guo et al. (2014); Di Stefano et al. (2019)
$k_0$	Reference permeability	2.17 $\times$ 10$^5$ $\hbox {mm}^2$ $\hbox {MPa}^{-1}$ $\hbox {day}^{-1}$	Basser (1992); Smith and Humphrey (2007); Jin et al. (2016); Asgari et al. (2016)

To complete the parameters overview, we need to provide a definition for the diffusion tensor $\mathbb {D}$ and for the tensor of preferential directions $\mathbb {A}$, influencing the permeability $\mathbb {K}$. To do so, we take advantage of the mechanical description included in the present model to progressively modify these tensors as time evolves. Indeed, the unnatural displacement induced by the neoplasm alters the direction of brain fibres in the surroundings, which should be taken into account in the description of both diffusion and fluid motion. It has been experimentally observed, by analysing both the DTI and the MRI scans of glioma patients, that volumetric and diffusion alterations can be recorded not only in the tumour region, but also in the surrounding healthy tissue, confirming structural and connectivity abrasions of brain areas distant from the brain tumour, and providing insights into the pathogenesis of diverse neurological symptoms in glioma patients (Bouwen et al. 2018). Since our model explicitly evaluates the deformation and the displacement caused by the tumour, we are able to track these changes and to exploit them to modify tissue anisotropy.

In detail, we start from a diffusion tensor $\mathbb {D}_0$ considered at the initial time instant, which can be inferred directly from DTI imaging data after a proper computational processing described in Sect. 3. Since we consider oxygen as the main nutrients source, it seems appropriate to employ these data in the nutrients balance equation, given that the DTI scan actually quantifies the diffusion of water inside the brain. Then, we can write

26 $$\begin{aligned} \mathbb {D}_0 = \lambda _1 \mathbf{e} _1^0 \otimes \mathbf{e} _1^0 + \lambda _2 \mathbf{e} _2^0 \otimes \mathbf{e} _2^0 + \lambda _3 \mathbf{e} _3^0 \otimes \mathbf{e} _3^0 \, , \end{aligned}$$

where we have put in evidence the descending order eigenvalues $\lambda _1> \lambda _2 > \lambda _3$ and the corresponding orthogonal eigenvectors $\mathbf{e} _1^0, \mathbf{e} _2^0, \mathbf{e} _3^0$.

Concerning $\mathbb {A}_0$, i.e. the initial value of tensor $\mathbb {A}$, its construction is also performed using DTI data, to evaluate the preferential directions identified by the presence of white matter tracts. In particular, it is assumed that $\mathbb {A}_0$ has the same eigenvectors as the diffusion tensor, but increased anisotropy along the preferential directions of motion inside the brain, as described in Jbabdi et al. (2005), Agosti et al. (2018a, b). To enhance anisotropy without altering the preferred directions, a control parameter *r* is introduced and $\mathbb {A}_0$ is defined as

27 $$\begin{aligned}&\widehat{\mathbb {A}}_0 = a_1(r) \lambda _1 \mathbf {e}_1^0 \otimes \mathbf {e}_1^0 + a_2(r) \lambda _2 \mathbf {e}_2^0 \otimes \mathbf {e}_2^0 +\lambda _3 \mathbf {e}_3^0 \otimes \mathbf {e}_3^0, \\&\mathbb {A}_0 = \frac{1}{\text {A}_{av}^0} \widehat{\mathbb {A}}_0 \, , \qquad \text {A}_{av}^0 = \frac{1}{3} {{\,\mathrm{tr}\,}}(\widehat{\mathbb {A}}_0). \end{aligned}$$

In the previous expressions, *r* is the tuning parameter of anisotropy and $a_i(r)$ are functions of *r* given by

28 $$\begin{aligned} \begin{aligned}&{ a_1(r) = r \mathrm {a}_l + r \mathrm {a}_p + \mathrm {a}_s \, , } \\&{ a_2(r) = \mathrm {a}_l + r \mathrm {a}_p + \mathrm {a}_s \, , } \end{aligned} \end{aligned}$$

where the coefficients $\mathrm {a}_l$, $\mathrm {a}_p$, $\mathrm {a}_s$ are the *linear*, *planar* and *spherical* anisotropy indices, respectively, defined as (Jbabdi et al. 2005; Westin et al. 2002; Painter and Hillen 2013):

29 $$\begin{aligned} \mathrm {a}_l= \frac{\lambda _1 - \lambda _2}{\lambda _1 + \lambda _2 + \lambda _3} \, , \quad \mathrm {a}_p = \frac{2(\lambda _2 - \lambda _3)}{\lambda _1 + \lambda _2 + \lambda _3} \, , \quad \mathrm {a}_s = \frac{3 \lambda _3}{\lambda _1 + \lambda _2 + \lambda _3} \, , \end{aligned}$$

with $\lambda _k$, $k=1,2,3$, being the eigenvalues of the diffusion tensor considered in decreasing order. The definition of these coefficients stems from the three simplest modes of diffusion: indeed, when $\lambda _1 \gg \lambda _2 \approx \lambda _3$, then $\mathrm {a}_l \approx 1$ and diffusion preferentially happens linearly along the direction of $\mathbf{e }_1^0$. On the other hand, if $\lambda _1 \approx \lambda _2 \gg \lambda _3$, the diffusion process is mainly confined into the plane spanned by $\mathbf{e }_1^0$ and $\mathbf{e }_2^0$, leading to the planar coefficient $\mathrm {a}_p \approx 1$. Finally, in the case of spherical diffusion, all the eigenvalues of $\mathbb {D}_0$ have the same order of magnitude and $\mathrm {a}_s \approx 1$. Since, in general, the diffusion tensor will feature a combination of all these modes, it can be decomposed (Westin et al. 2002) as

$$\begin{aligned} { \mathbb {D}_0 = (\lambda _1 - \lambda _2)\mathbb {D}_l + (\lambda _2 - \lambda _3)\mathbb {D}_p + \lambda _3 \mathbb {D}_s \, , } \end{aligned}$$

where $\mathbb {D}_l := \mathbf{e }_1^0 \otimes \mathbf{e }_1^0$, $\mathbb {D}_p := \mathbf{e }_1^0 \otimes \mathbf{e }_1^0 + \mathbf{e }_2^0 \otimes \mathbf{e }_2^0$ and $\mathbb {D}_s := \mathbf{e }_1^0 \otimes \mathbf{e }_1^0 + \mathbf{e }_2^0 \otimes \mathbf{e }_2^0 + \mathbf{e }_3^0 \otimes \mathbf{e }_3^0$. Therefore, the coefficients $\mathrm {a}_l$, $\mathrm {a}_p$, $\mathrm {a}_s$ are related to the components of $\mathbb {D}_0$ with respect to the tensor basis $\{\mathbb {D}_l, \mathbb {D}_p, \mathbb {D}_s\}$: the scaling factors and trace normalization are introduced to guarantee that the coefficients range between 0 and 1, while keeping their sum equal to one. Concerning the definition of the anisotropy coefficients appearing in Eq. (28), they are employed to introduce changes in anisotropy through the parameter *r*, as done in Jbabdi et al. (2005). In particular, the case $r = 1$ corresponds to no increase in anisotropy, since $\mathrm {a}_l + \mathrm {a}_p + \mathrm {a}_s = 1$, while $r > 1$ enhances anisotropy along the directions of the eigenvectors according to the values of the coefficients of anisotropy.

**Fig. 2:**
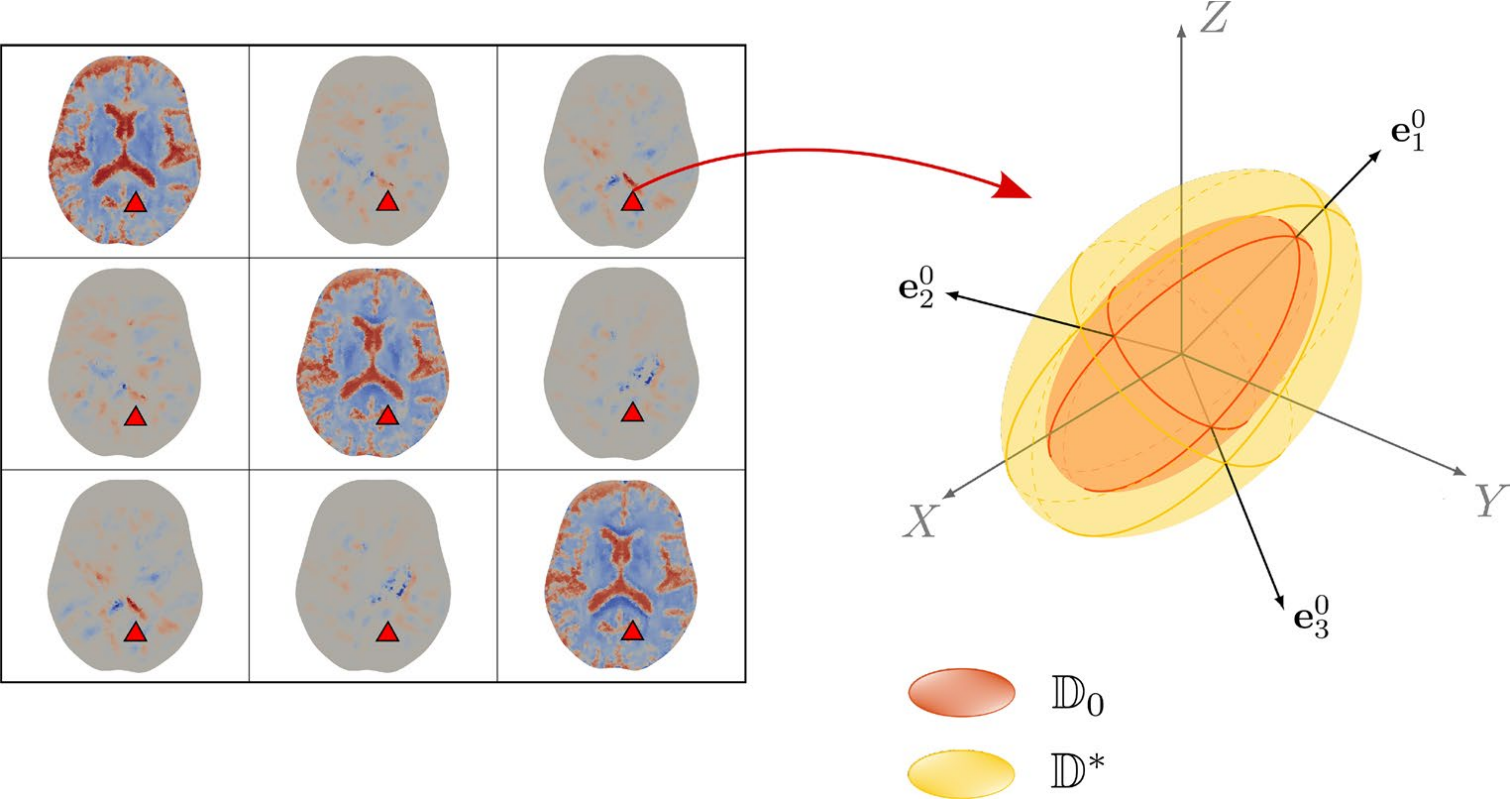
Computational reconstruction and modification of the components of the diffusion tensor taken from DTI data. The initial tensor $\mathbb {D}_0$ is built using medical imaging data (as explained in Sect. 3) and the values of the six components of the symmetric diffusion tensor are assigned to each mesh cell. In particular, on the left we show a sample of the components of the tensor $\mathbb {D}_0$ as they appear on a transverse (horizontal) brain section, with higher values of the diffusion coefficients plotted in red. For a fixed representative cell sketched by the red triangle, on the right, we draw the red ellipsoid representing the preferential directions and values of diffusion at the initial time instant, i.e. the eigenvectors and eigenvalues of $\mathbb {D}_0$, respectively. The initial eigenvectors are modified according to the deformation of the tissue, in order to obtain the time and spatially dependent tensor $\mathbb {D}$, given by Eq. (30). In the figure, we report $\mathbb {D}_0$ and the pullback $\mathbb {D}^* := J_{\mathrm{s}} \mathbb {F}_{\mathrm{s}}^{-1}\mathbb {D}\mathbb {F}_{\mathrm{s}}^{\mathrm{-T}}$ of the modified diffusion tensor (in yellow), which are both defined in the reference configuration. We observe that $\mathbb {D}^*$ has the same eigenvectors as $\mathbb {D}_0$ but different eigenvalues, due to the normalization and volumetric changes

Once we have built the starting tensors $\mathbb {D}_0$ and $\mathbb {A}_0$, their modification subsequent to growth and deformation is done taking into account that, as far as diffusion is concerned, it is relevant to consider just the reorientation of the preferential directions and not their extension or compression that, in principle, does not affect nutrients diffusion and cell motility. Therefore, we deform the eigenvectors according to the deformation gradient $\mathbb {F}_{\mathrm{s}}$, but we normalize them to account for the fact that only the direction of the fibres is changing (see Fig. 2). Hence, for the modified diffusion tensor we write

30 $$\begin{aligned} \mathbb {D}= \lambda _1 \frac{\mathbb {F}_{\mathrm{s}}\mathbf{e }_1^0 \otimes \mathbb {F}_\mathrm{s}\mathbf{e }_1^0}{|\mathbb {F}_{\mathrm{s}}\mathbf{e }_1^0|^2} + \lambda _2 \frac{\mathbb {F}_\mathrm{s}\mathbf{e }_2^0\otimes \mathbb {F}_{\mathrm{s}}\mathbf{e }_2^0}{|\mathbb {F}_{\mathrm{s}}\mathbf{e }_2^0|^2} + \lambda _3 \frac{\mathbb {F}_{\mathrm{s}}\mathbf{e }_3^0 \otimes \mathbb {F}_\mathrm{s}\mathbf{e }_3^0}{|\mathbb {F}_{\mathrm{s}}\mathbf{e }_3^0|^2} \, , \end{aligned}$$

where we observe that

$$\begin{aligned} \left| \mathbb {F}_{\mathrm{s}}\mathbf{e }_i^0\right| ^2 = \mathbb {F}_{\mathrm{s}}\mathbf{e }_i^0 \cdot \mathbb {F}_{\mathrm{s}}\mathbf{e }_i^0 = \mathbf{e }_i^0 \cdot \mathbb {C}_{\mathrm{s}}\mathbf{e }_i^0 \, , \quad i = 1,2,3, \quad \mathbb {C}_{\mathrm{s}} = \mathbb {F}_{\mathrm{s}}^{\mathrm{T}} \mathbb {F}_{\mathrm{s}} . \end{aligned}$$

We remark that, in defining the modified diffusion tensor, we choose to keep unchanged the trace of the initial tensor, and therefore the mean diffusivity along the principal directions. As a consequence of these assumptions, the pullback $\mathbb {D}^*$ of the modified diffusion tensor $\mathbb {D}$ to the reference configuration does not coincide with $\mathbb {D}_0$ and the volume of the diffusion ellipsoid is not preserved, in general (see Fig. 2).

The modified tensor of preferential directions $\mathbb {A}$ can be defined using the same procedure as

31 $$\begin{aligned}&\widehat{\mathbb{A}} = a_1(r)\lambda _1 \frac{\mathbb {F}_{\mathrm{s}}\mathbf{e }_1^0 \otimes \mathbb {F}_{\mathrm{s}}\mathbf{e }_1^0}{\mathbf{e }_1^0\cdot \mathbb {C}_{\mathrm{s}}\mathbf{e }_1^0} + a_2(r)\lambda _2 \frac{\mathbb {F}_{\mathrm{s}}\mathbf{e }_2^0\otimes \mathbb {F}_{\mathrm{s}}\mathbf{e }_2^0}{\mathbf{e }_2^0\cdot \mathbb {C}_{\mathrm{s}}\mathbf{e }_2^0} + \lambda _3 \frac{\mathbb {F}_{\mathrm{s}}\mathbf{e }_3^0 \otimes \mathbb {F}_{\mathrm{s}}\mathbf{e }_3^0}{\mathbf{e }_3^0\cdot \mathbb {C}_{\mathrm{s}}\mathbf{e }_3^0}, \\&\mathbb{A} = \frac{1}{\text {A}_{av}} \widehat{\mathbb {A}} \, , \qquad \text {A}_{av} = \frac{1}{3} {{\,\mathrm{tr}\,}}(\widehat{\mathbb {A}}).\end{aligned}
$$

## Materials and methods

To perform simulations and solve our equations numerically, we need to introduce a spatially and temporally discrete formulation of the continuous problem. Therefore, in this Section we describe the procedures to generate the patient-specific mesh used for the computation and the additional meshes containing the values of the components of the diffusion tensor $\mathbb {D}_0$ and of the tensor of preferential directions $\mathbb {A}_0$, built starting from MRI and DTI clinical data of a patient affected by a brain tumour (a GBM, specifically), gently provided by the Istituto Neurologico Carlo Besta (Milan, Italy). We then introduce the Lagrangian formulation of the model, that allows to solve the problem in the reference configuration, and finally we report the finite element and time discretization of the problem.

### Mesh creation and preprocessing

**Fig. 3:**
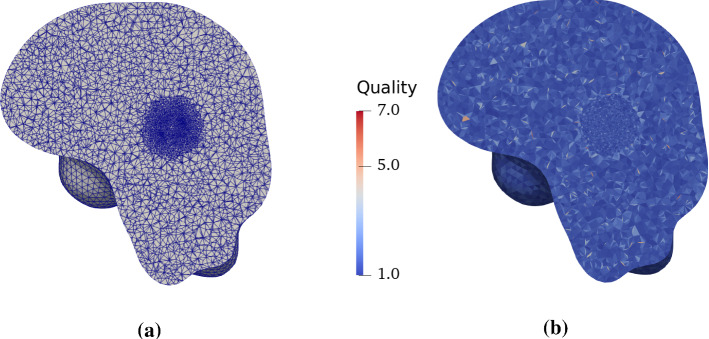
**a** Tetrahedral mesh generated within the brain domain, reconstructed from neuroimaging data, and properly refined in the tumour region; **b** mesh quality in terms of tetrahedral aspect ratio

A mesh generation pipeline is constructed using the analysis tools provided by the FSL software library (Woolrich et al. 2009) for image processing and segmentation and by the VMTK software library (Antiga et al. 2008) for mesh generation. In particular, starting from a T1-w MR image at 1 mm $\times$ 1 mm $\times$ 1 mm spatial resolution, which provides the structural anatomy of the patient’s brain, the following preprocessing and computational steps are performed:

- brain extraction by intensity thresholding, in order to remove non-brain tissues, and bias-field correction;
- segmentation of the brain tissues and the background using the FAST algorithm (Zhang et al. 2001), based on a hidden Markov random field model and an associated expectation–maximization algorithm to estimate the segmentation maps;
- extraction of a polygonal mesh of the isosurface representing the external brain boundary from the segmentation maps, using the marching cubes algorithm (Lorensen and Cline 1987). Further application of surface smoothing, using Taubin’s algorithm (Taubin 1995), and refinement steps to the isosurface mesh are implemented.
- Generation of the 3D mesh using a constrained Delaunay tetrahedralization of the brain domain defined by its boundary using the TetGen library (Si 2015), with proper refinement by smooth sizing functions in the area surrounding the tumour centre.

The resulting computational brain mesh and its quality evaluated in terms of aspect ratio are reported in Fig. 3. In particular, we verified that almost all elements ($>95\%$) had an aspect ratio smaller than five.

A diffusion reconstruction pipeline is further defined using the ANIMA toolbox. In particular, starting from raw diffusion data from a DTI sequence comprising a set of 140 diffusion-weighted images at 2 mm $\times$ 2 mm $\times$ 2 mm spatial resolution with anterior–posterior phase encoding direction with different b-values and diffusion-sensitizing directions, with an additional image acquired with reversed phase encoding direction, the following preprocessing and reconstruction steps are performed:

- correction of the eddy current distortion (i.e. the artefacts in the DTI images due to the electrical currents induced by the rapid switching of the magnetic field gradients during DTI acquisition) by nonlinear registration, computed in the phase encoding direction, of the diffusion images onto a single diffusion image;
- estimation of the susceptibility distortion field from the forward and backward phase encoding images, with correction of the susceptibility-induced deformations by symmetric block-matching nonlinear registration (Hédouin et al. 2017);
- denoising of each diffusion image by blockwise non-local means filtering (Coupe et al. 2008);
- brain masking, obtained by block-matching rigid registration of the brain mask extracted from the T1–w image;
- estimation of the tensor $\mathbb {D}_0$ of water diffusion in each voxel from the diffusion images by matrix inversion using the log-signals, assuming that water diffuses according to a Gaussian process with zero mean and covariance proportional to the diffusion tensor (Arsigny et al. 2006);
- projection of the estimated tensor onto the space of the T1-w image by affine block-matching registration.

We finally generate six additional meshes, each one with a piecewise constant field associated with one independent component of the tensor $\mathbb {D}_0$, by assigning to each cell the value of the tensor component of the voxel containing the cell barycentre, as shown in Fig. 2. At the same time, additional six meshes are also associated with each independent component of the tensor of preferential directions $\mathbb {A}_0$, expressed in terms of the eigenvalues and eigenvectors of the diffusion tensor $\mathbb {D}_0$ as explained in Sect. 2.2.

### Numerical implementation

#### Lagrangian formulation

Since we solve our equations in the reference configuration, we rewrite the model in material coordinates. In the following, unless otherwise specified, we will use the same symbols to denote the variables in the spatial and material description, and also omit the explicit spatial dependence. Instead, we decide to employ a different notation to distinguish between differential operators acting on different configurations: henceforth, Grad and Div will be used to denote the material gradient and material divergence, respectively, i.e. gradient and divergence with respect to the material point $\mathbf {X}$ in the reference configuration.

By classical computations (Ambrosi and Mollica 2002; Grillo et al. 2012; Mascheroni et al. 2018; Giverso and Preziosi 2019), using a superimposed dot to denote the material time derivative, it is possible to derive the following complete set of equations, holding in the fixed reference domain $\Omega ^*$:

32a $$\begin{aligned}&J_{\mathrm{s}} = \det \mathbb {F}_{\mathrm{s}} \, , \qquad \mathbb {F}_{\mathrm{s}} = \mathbb {I} + {{\,\mathrm{Grad}\,}}\mathbf {u}_{\mathrm{s}} \, , \end{aligned}$$

32b $$\begin{aligned}&J_{\mathrm{s}} \phi _{\mathrm{s}} = J_{\mathrm{g}} \phi _{\mathrm{sn}} \, , \qquad \phi _{\mathrm{s}} + \phi _{\ell } = 1 \, , \end{aligned}$$

32c $$\begin{aligned}&{\dot{J}}_{\mathrm{s}} = {{\,\mathrm{Div}\,}}\left[ \frac{\mathbb {K}^*}{\mu } {{\,\mathrm{Grad}\,}}p \right] \, , \end{aligned}$$

32d $$\begin{aligned}&{{\,\mathrm{Div}\,}}\left[ -J_{\mathrm{s}} p \mathbb {F}_{\mathrm{s}}^{\mathrm{-T}} + \mathbb {P}_{\mathrm{s}} \right] = \mathbf {0} \, ,\end{aligned}$$

32e $$\begin{aligned}&{\dot{g}} = g \frac{\Gamma _{\mathrm{s}}}{3 \phi _{\mathrm{s}}} \chi _{\Omega _{\mathrm{t}}^*} \, , \end{aligned}$$

32f $$\begin{aligned}&J_{\mathrm{s}} \phi _{\ell } {\dot{c}}_n - \frac{\mathbb {K}^*}{\mu } {{\,\mathrm{Grad}\,}}p \cdot {{\,\mathrm{Grad}\,}}c_n - {{\,\mathrm{Div}\,}}\left[ \phi _{\ell } \mathbb {D}^* {{\,\mathrm{Grad}\,}}c_n \right] = J_{\mathrm{s}} G_n \chi _{\Omega _{\mathrm{t}}^*} , \end{aligned}$$

where $\mathbb {K}^* := J_{\mathrm{s}} \mu k(J_{\mathrm{e}}) \mathbb {F}_\mathrm{s}^{-1} \mathbb {A} \mathbb {F}_{\mathrm{s}}^{\mathrm{-T}}$, $\mathbb {D}^* := J_{\mathrm{s}} \mathbb {F}_{\mathrm{s}}^{-1} \mathbb {D} \mathbb {F}_{\mathrm{s}}^\mathrm{-T}$ and $\mathbb {D}$, $\mathbb {A}$ are the tensors modified according to the deformation, as reported in Eqs. (30)-(31). We remark that the pullbacks $\mathbb {D}^*$ and $\mathbb {A}^* = J_\mathrm{s}\mathbb {F}^{-1}\mathbb {A}\mathbb {F}^{-\mathrm T}$ of $\mathbb {D}$ and $\mathbb {A}$ have the same eigenvectors as their initial counterparts $\mathbb {D}_0$ and $\mathbb {A}_0$, but the eigenvalues are rescaled because of the normalization of the deformed eigenvectors and volumetric changes (see Eq. (30–31) and Fig. 2). Instead, $\mathbb {P}_{\mathrm{s}}$ is the constitutive part of the first Piola–Kirchhoff stress tensor of the solid phase, $\mathbb {P}_\mathrm{s} = J_{\mathrm{s}} \mathbb {T}_{\mathrm{s}} \mathbb {F}_{\mathrm{s}}^{\mathrm{-T}}$. We notice that the system (32) is closed, since it constitutes a set of eight scalar equations (32b)-(32f) and features eight scalar unknowns, namely the displacement vector field of the solid phase $\mathbf {u}_{\mathrm{s}}(\mathbf {X},t)$ and the scalar fields $\phi _{\mathrm{s}}(\mathbf {X},t)$, $\phi _{\ell }(\mathbf {X},t)$, $g(\mathbf {X},t)$, $c_n(\mathbf {X},t)$ and $p(\mathbf {X},t)$. The fluid velocity $\mathbf {v}_{\ell }(\mathbf {X},t)$ can be derived using Eq. (13). We remark that, since all the equations are pulled back on the reference configuration using the deformation field of the solid phase, the indicator function $\chi _{\Omega _{\mathrm{t}}^*}$ does not evolve in space and time, so it is not an additional unknown for the model and an evolution equation is not needed.

The system of equations (32) allows therefore to determine all the unknown fields, $\forall \mathbf {X} \in \Omega ^*$ and $\forall t \in (0,T)$, provided that proper initial and boundary conditions are prescribed. Since in our simulations the external boundary $\partial \Omega ^*$ stands for the cranial skull, we consider the following set of boundary conditions:

33a $$\begin{aligned} \mathbf {u}_{\mathrm{s}} = \mathbf {0} \qquad&\text { on } \qquad \partial \Omega ^*, \forall \, t \in (0,T) \, , \end{aligned}$$

33b $$\begin{aligned} p = 0 \qquad&\text { on } \qquad \partial \Omega ^*, \forall \, t \in (0,T) \, , \end{aligned}$$

33c $$\begin{aligned} c_n = 1 \qquad&\text { on } \qquad \partial \Omega ^*, \forall \, t \in (0,T) \, . \end{aligned}$$

In detail, we impose a null Dirichlet boundary condition for the displacement $\mathbf {u}_{\mathrm{s}}$ and for the pressure *p* at the boundary of the cranial skull. As regards the nutrients concentration, we suppose that the brain boundary is sufficiently far from the tumour: we can then assume that, on the boundary, the oxygen concentration is maintained constant at the physiological value of 1. When the cancer grows close to the skull, as shown for instance in Supplementary Figures S1–S5, zero-flux boundary conditions for the pressure and for the nutrients concentration might be more appropriate. For a more detailed discussion, we refer the reader to the Supplementary Material of the article.

Concerning initial conditions, at the beginning of the tumour growth process it is reasonable to assume that the displacement and the pressure are equal to zero everywhere in the domain; meanwhile, we take the scalar field *g* related to the growth component of the deformation gradient as equal to 1 everywhere in the domain at $t = 0$.

Enforcing the condition that the variation of body mass is due to cell proliferation, it is possible to show (Ambrosi and Mollica 2002; Grillo et al. 2012) that the solid volumetric fraction in the natural state $\phi _{\mathrm{sn}}$ is constant in time and, thus, equal to the $\phi _{\mathrm{s}}(\mathbf {X}, 0)$. In particular, in the following we will consider $\phi _{\mathrm{sn}}$ homogeneous in space. Finally the initial nutrients concentration is uniformly set to $c_n = 1$ everywhere. To sum up, we have the following set of initial conditions:

34a $$\begin{aligned} \mathbf {u}_{\mathrm{s}}(\mathbf {X}, 0) = \mathbf {0} \qquad&\forall \, \mathbf {X} \in \Omega ^* \, ,\end{aligned}$$

34b $$\begin{aligned} p(\mathbf {X}, 0) = 0 \qquad&\forall \, \mathbf {X} \in \Omega ^* \, , \end{aligned}$$

34c $$\begin{aligned} g(\mathbf {X}, 0) = 1 \qquad&\forall \, \mathbf {X} \in \Omega ^* \, ,\end{aligned}$$

34d $$\begin{aligned} \phi _{\mathrm{s}}(\mathbf {X}, 0) = \phi _{\mathrm{sn}} \qquad&\forall \, \mathbf {X} \in \Omega ^* \, ,\end{aligned}$$

34e $$\begin{aligned} c_n(\mathbf {X}, 0) = 1 \qquad&\forall \, \mathbf {X} \in \Omega ^* \,. \end{aligned}$$

#### Finite element discretization

To perform numerical simulations, we here introduce the spatially discrete formulation of a proper continuous variational formulation of the system (32). We make use of linear tetrahedron $\mathbb {P}_1$ elements, so we introduce the following finite element spaces:

35 $$\begin{aligned}&\varvec{V}_h := \{ \varvec{q}_h \in \left[ C^0(\overline{\Omega ^*})\right] ^3 : \varvec{q}_h|_{K} \in \left[ \mathbb {P}_1(K)\right] ^3 \;\; \forall \, K \in \mathcal {T}_h \, , \; \varvec{q}_h|_{\partial \Omega ^*} = \varvec{0} \} \subset \varvec{H^1_0}(\Omega ^*) \, , \end{aligned}$$

36 $$\begin{aligned}&W_{h0} := \{ q_h \in C^0(\overline{\Omega ^*}) : q_h|_{K} \in \mathbb {P}_1(K) \;\; \forall \, K \in \mathcal {T}_h \, , \; q_h|_{\partial \Omega ^*} = 0 \} \subset H^1_0(\Omega ^*) \, , \end{aligned}$$

37 $$\begin{aligned}&W_{h1} := \{ q_h \in C^0(\overline{\Omega ^*}) : q_h|_{K} \in \mathbb {P}_1(K) \;\; \forall \, K \in \mathcal {T}_h \, , \; q_h|_{\partial \Omega ^*} = 1 \} \subset H^1(\Omega ^*) \, , \end{aligned}$$

where $\mathcal {T}_h$ is a conforming decomposition of the domain $\Omega ^*$ into tetrahedra *K*. Then, we are able to define our fully discrete variational problem as follows: for $k = 1, \dots , N$, given $(\mathbf {u}_{h}^k, p_h^k, c_{h}^k)~ \in ~\varvec{V}_h \times W_{h0} \times W_{h1}$ find $(\mathbf {u}_{h}, p_h, c_{h}) \in \varvec{V}_h \times W_{h0} \times W_{h1}$ such that $\forall \, (\varvec{v}_{h}, w_{h}, q_{h}) \in \varvec{V}_h \times W_{h0} \times W_{h0}$

38 $$\begin{aligned}&\left( \, J_{\mathrm{s}}(\mathbf {u}_{h}), w_{h} \, \right) + \Delta t \,\, \left( {{\,\mathrm{Grad}\,}}w_{h}, \frac{\mathbb {K}^*}{\mu } {{\,\mathrm{Grad}\,}}p_h\right) = \left( \, J_{\mathrm{s}}^k(\mathbf {u}_{h}^k), w_{h} \, \right) , \end{aligned}$$

39 $$\begin{aligned}&{- \left( \, \mathbb {P}(\mathbf {u}_{h}, p_h) , {{\,\mathrm{Grad}\,}}\varvec{v}_{h} \, \right) = \varvec{0}} \, , \end{aligned}$$

40 $$\begin{aligned}&\left( \, J_{\mathrm{s}}(\mathbf {u}_{h}) c_h, q_h \, \right) - \Delta t \left( \, \frac{\mathbb {K}^*}{\mu \phi _{\ell }} {{\,\mathrm{Grad}\,}}p_h \cdot {{\,\mathrm{Grad}\,}}c_h, q_h \, \right) \nonumber \\&\quad + \Delta t \left( \, {{\,\mathrm{Grad}\,}}q_h, \mathbb {D}^* {{\,\mathrm{Grad}\,}}c_h \, \right) \\&\quad = \left( \, J_\mathrm{s}(\mathbf {u}_h) c_h^k, q_h \, \right) + \Delta t \left( \, J_\mathrm{s}(\mathbf {u}_h) \, \frac{G_n(c_h)}{\phi _{\ell }}, q_h \, \right) ,\end{aligned}$$

where, in order to have a lighter notation, we have dropped the unnecessary superscripts and denoted by the same symbol $(\cdot , \cdot )$ the standard scalar product on the spaces $L^2(\Omega ^*)$, $L^2(\Omega ^*;\mathbb {R}^3)$ and $L^2(\Omega ^*;\mathbb {R}^{3\times 3})$. Finally, we have indicated by $\mathbb {P} = -J_{\mathrm{s}} p \mathbb {F}_{\mathrm{s}}^{- \mathrm T} + \mathbb {P}_{\mathrm{s}}$ the first Piola–Kirchhoff stress tensor. We remark that, since we are working in a Lagrangian configuration, also the tensors $\mathbb {K}^*$ and $\mathbb {D}^*$ depend on $\mathbf {u}_h$. Moreover, the time discretization in Eqs. (38)–(40) consists in a semi-implicit Euler scheme, which is solved by a Newton’s method. Since it is not evident how to define a single CFL condition (Courant et al. 1967) for the whole numerical problem, we chose a sufficiently small time step of $\Delta t = 0.1$ days and we checked, in the numerical code, that our choice guarantees the fulfilment of the CFL condition at each iteration.

Once we have obtained the discrete formulation of the partial differential equations, the last step is to introduce a proper discretization of the other equations involved, namely the ordinary differential equation for *g* (32e) and the relations (32b).

Regarding (32e), it can be easily discretized in time using the explicit Euler method. Then, we have

41 $$\begin{aligned} g^{k+1}(\mathbf {X}_j) = g^k(\mathbf {X}_j) \left( 1+ \Delta t \, \frac{\Gamma _{\mathrm{s}}^{k}(\mathbf {X}_j)}{3\, \phi _\mathrm{s}^{k}(\mathbf {X}_j)} \chi _{\Omega _{\mathrm{t}}^*} \right) \qquad j = 1,\dots ,M \end{aligned}$$

where $\mathbf {X}_j$ are the grid nodes and *M* is the number of spatial nodes in the discretization. The first equation of (32b) is simply discretized as follows

42 $$\begin{aligned}&J_{\mathrm{s}}^{k+1}(\mathbf {X}_j) \phi _{\mathrm{s}}^{k+1}(\mathbf {X}_j) = J_{\mathrm{g}}^{k+1}(\mathbf {X}_j) \phi _{\mathrm{sn}} \nonumber \\&\quad \Rightarrow \qquad \phi _{\mathrm{s}}^{k+1}(\mathbf {X}_j) = \frac{J_{\mathrm{g}}^{k+1}(\mathbf {X}_j)}{J_{\mathrm{s}}^{k+1}(\mathbf {X}_j)} \, \phi _{\mathrm{sn}} \, , \quad j=1,\dots ,M. \end{aligned}$$

Given the discretized form of all the necessary equations, we are now able to run numerical simulations of the model. To this end, we implemented our code using the open source computing software for solving partial differential equations called *FEniCS* (The FEniCS Project 2021; Alnaes et al. 2015; Logg et al. 2012). Such a software provides a high-level Python and C++ interface for solving PDEs through the finite element method: in particular, FEniCS code is attractive since it remains very close to the mathematical formulation, allowing the user to write down a program which closely resembles the variational form of equations. For instance, in FEniCS it is possible to choose the finite element of interest, define function spaces for test and trial functions, import external meshes easily and define a variational problem. It also comes with built-in classes specifically dedicated to the resolution of nonlinear variational problems, which in our case is an important feature. In particular, to solve the nonlinear variational problem defined by Eqs. (38)-(40), we employ the FEniCS built-in Newton’s method.

## Results

To test the model and its implementation, we perform some numerical simulations on a realistic brain geometry, constructed from medical imaging data following the procedure described in Sect. 3. As specified before, we use the finite element method to solve the equations: in particular, we consider an initial tumour radius of about 7 mm and simulate tumour progression for 45 days. In particular, in Sect. 4.1 we compare the results of tumour evolution using both the growth laws given in Eqs. (5) and (6), the latter including the effect of solid stress inhibition on cell proliferation. Then, in Sect. 4.2 we report the results concerning the modification of DTI data as a consequence of growth-induced deformations.

### Tumour evolution with and without stress inhibition

**Fig. 4:**
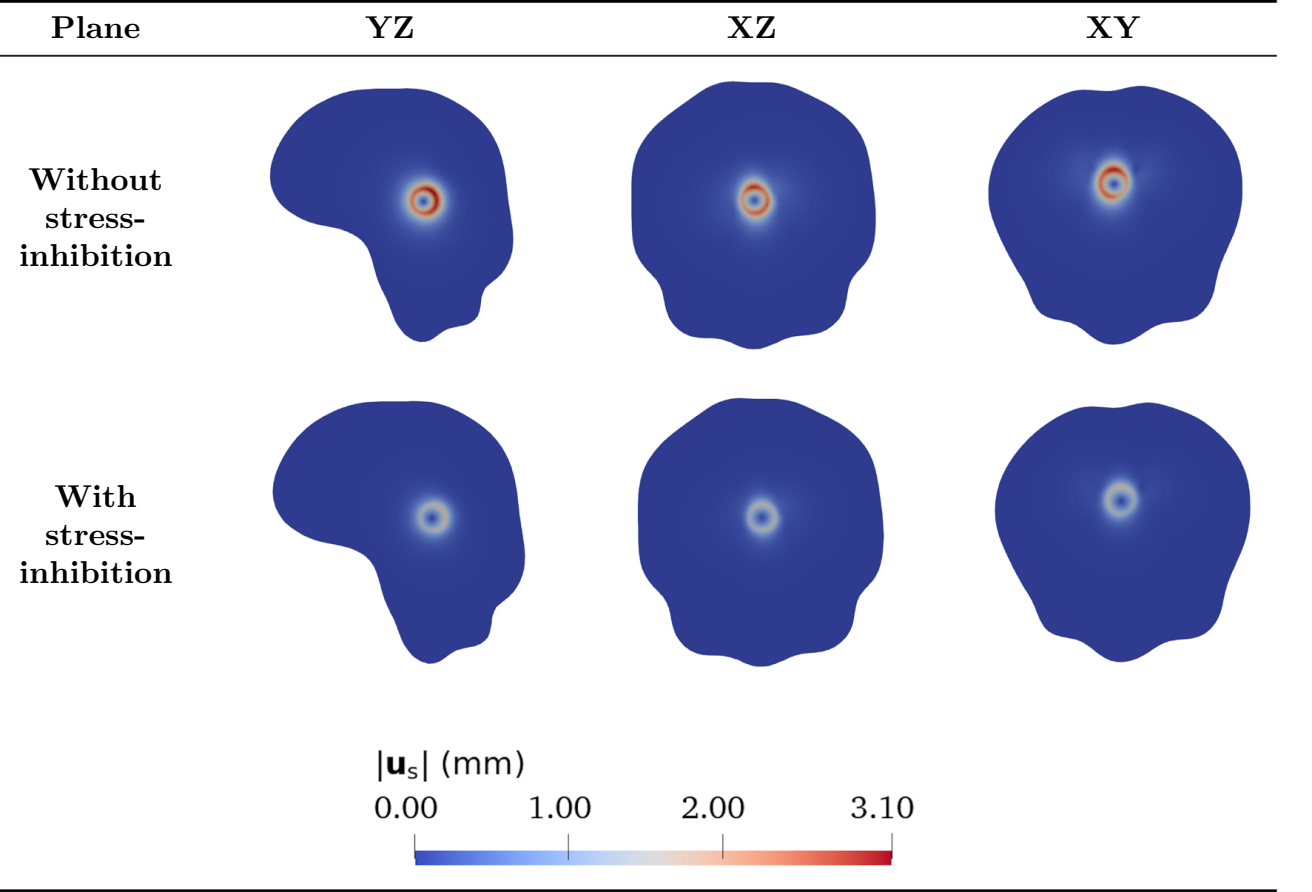
Comparison between the displacement magnitude $|\mathbf {u}_{\mathrm{s}}|$ after $t=45$ days of tumour growth in the brain, clipped along a sagittal (first column), an axial (second column), and a coronal (third column) plane centred within the tumour. In the first row, the case without stress inhibition is reported, while the second row shows a case of stress-inhibited growth. After a month and a half, the maximum displacement induced by the tumour without any inhibition due to stress amounts at 3.1 mm, while in the inhibited case it is about 1.7 mm

**Fig. 5:**
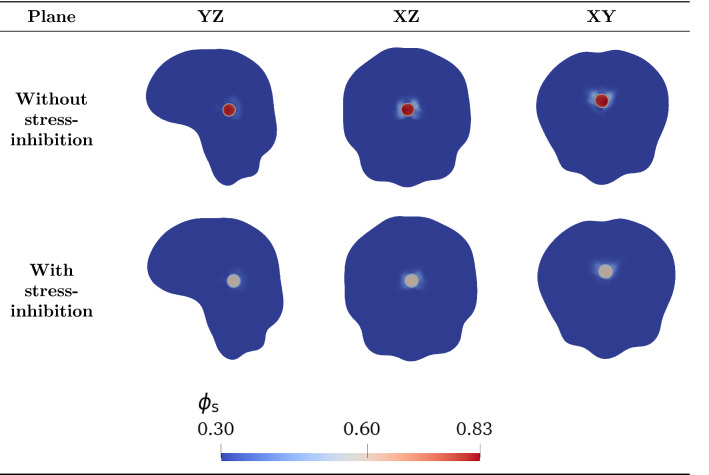
Comparison between the solid volume fraction $\phi _{\mathrm{s}}$ after $t=45$ days of tumour growth in the brain, clipped along a sagittal (first column), an axial (second column), and a coronal (third column) plane centred within the tumour. In the first row, the case without stress inhibition is reported, while the second row shows a case of stress-inhibited growth

**Fig. 6:**
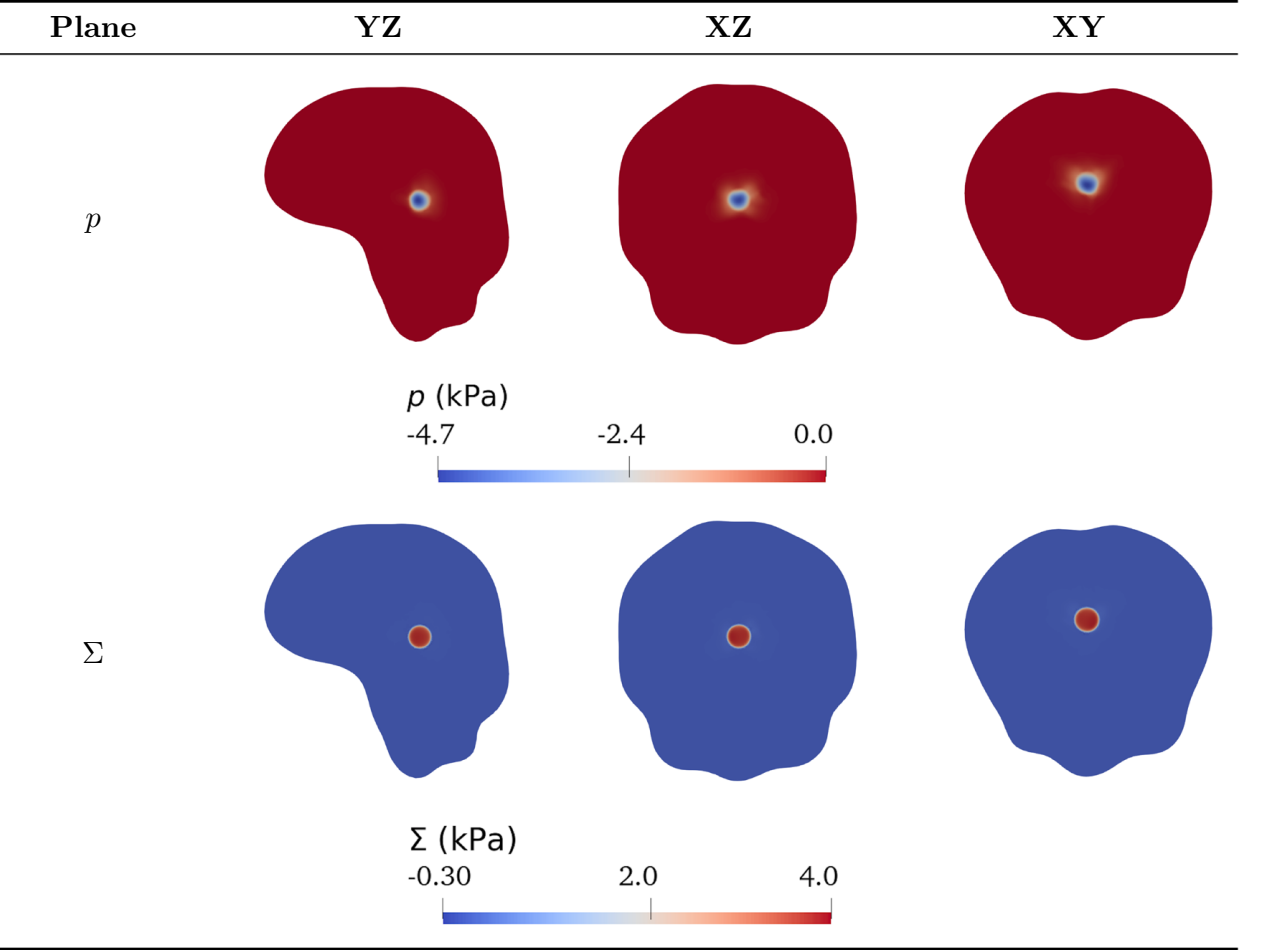
Comparison between variables during tumour growth in the brain, clipped along a sagittal (first column), an axial (second column), and a coronal (third column) plane centred within the tumour, at time $t = 45$ days. In the first row the fluid pressure *p* is reported, while the second row shows the stress measure $\Sigma = -\frac{1}{3}\text {tr}(\mathbb {T}_{\mathrm{s}})$

**Fig. 7:**
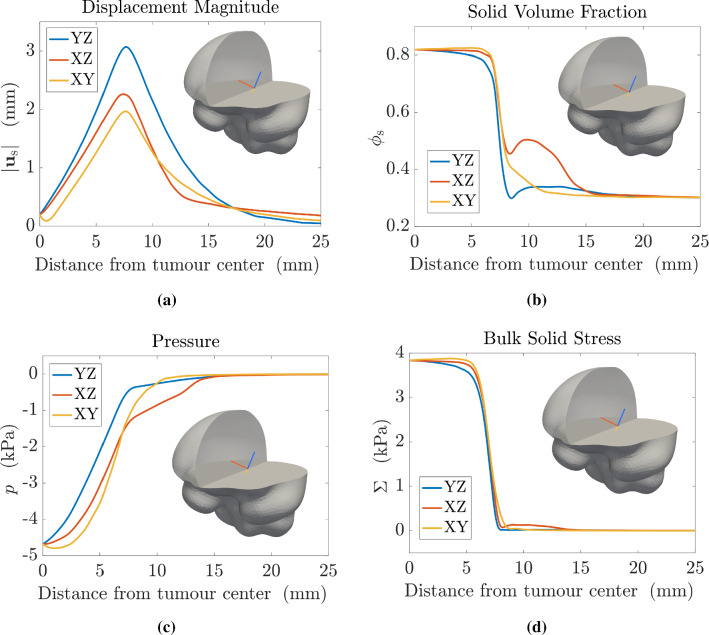
Comparison between variables during tumour growth in the brain, along three representative rays in different planes originating from the tumour centre, at time $t = 45$ days. In the insets, the chosen rays are depicted on the 3D brain mesh. **a** Displacement magnitude $|\mathbf{u} _{\mathrm{s}}|$; **b** solid volume fraction $\phi _{\mathrm{s}}$; **c** pressure *p*; **d** bulk solid stress $\Sigma$

**Fig. 8:**
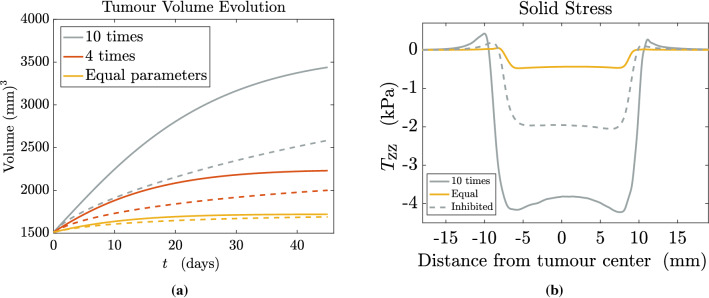
**a** Tumour volume evolution over time, for different values of the elastic parameters in the tumour region. In particular, the tumour is taken as ten times stiffer than the host tissue (grey curves), four times stiffer (red curves), and equal to the healthy tissue (yellow curves). Solid lines correspond to the cases without stress inhibition on growth, while dashed lines refer to the simulations with stress inhibition ($\delta _1 = 0.8$, $\delta _2 = 10^{-4}$ MPa). **b** $T_{\mathrm{zz}}$ component of the solid Cauchy stress tensor along a ray that crosses the tumour diameter, for the case of a tumour which is ten times stiffer (grey curves) and as stiff as the healthy tissue (yellow curve)

**Fig. 9:**
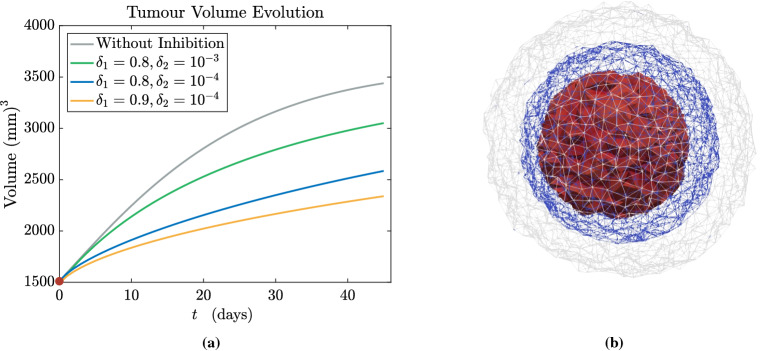
**a** Tumour volume evolution, as a function of time, in the case with and without stress inhibition of growth, for different values of the parameters $\delta _1$ and $\delta _2$ appearing in Eq. (6). The red marker identifies the initial volume of the tumour. **b** Initial tumour configuration (red), corresponding to the volume at $t=0$; final configuration for the case without stress inhibition (grey) and for the stress-inhibited case with $\delta _1 = 0.8$, $\delta _2 = 10^{-4}$ MPa (blue), corresponding to the final volumes in the line plot (**a**)

**Fig. 10:**
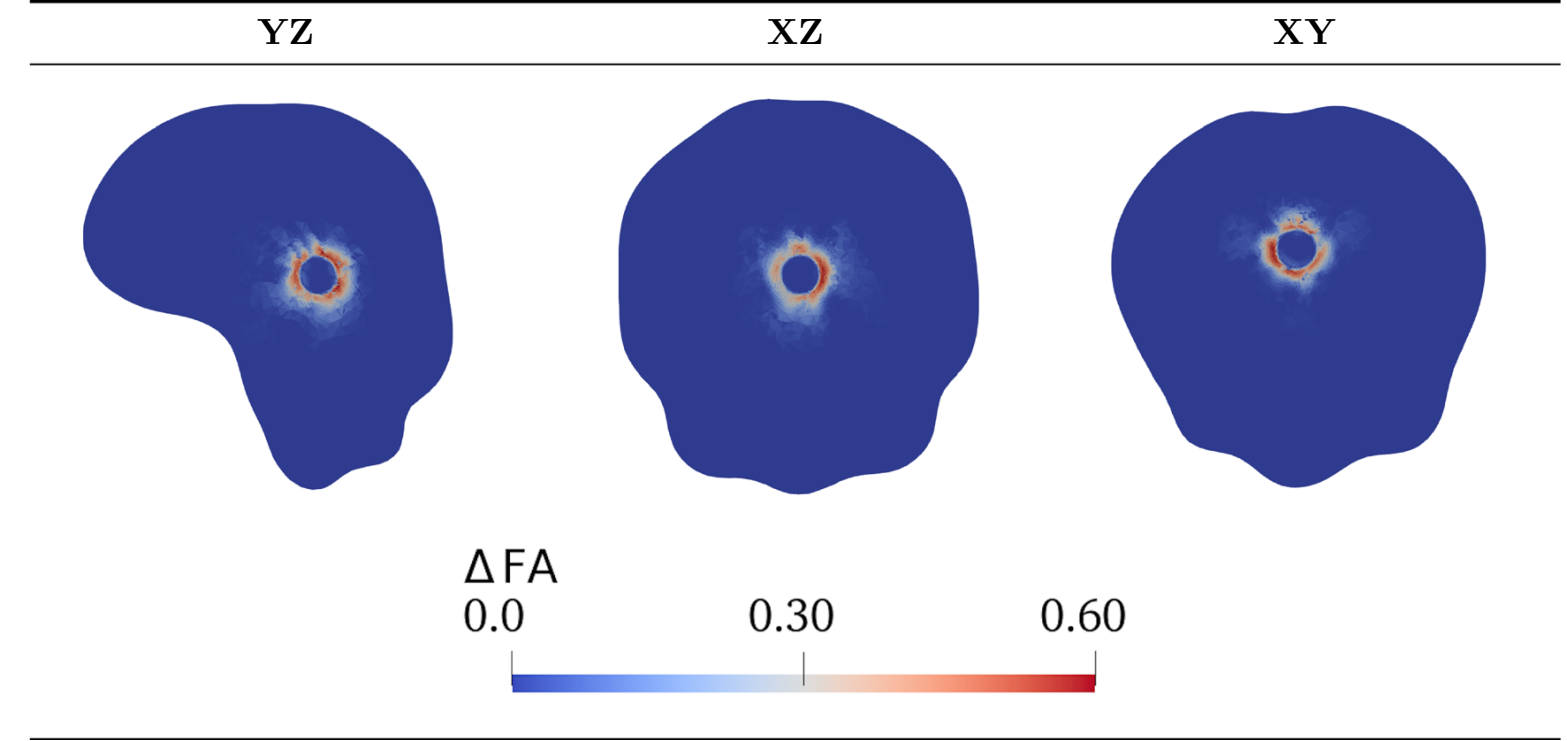
Variation of the fractional anisotropy (FA) after 45 days of tumour growth. It can be noted that, around the tumour zone, there is an increase in the tissue anisotropy

**Fig. 11:**
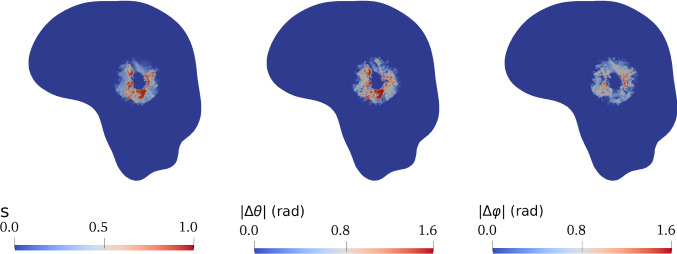
Variation during tumour growth (from $t=0$ to $t=45$ days) of the eigenvector associated with the greatest eigenvalue of the diffusion tensor $\mathbb {D}$, quantified in terms of the scalar index *s* (left) and the absolute variations of the azimuthal angle $|\Delta \theta |$ (centre) and polar angle $|\Delta \varphi |$ (right)

Results in terms of displacements, cell volume fraction, pressure, and the chosen measure of the stress are shown in Figs. 4, 5 and 6 along three sagittal, axial and coronal planes centred within the tumour. Specifically, in order to highlight the displacement induced by the growing mass, in Fig. 4 we report the magnitude $|\mathbf {u}_{\mathrm{s}}|$ of the displacement vector $\mathbf {u}_{\mathrm{s}}$. The comparison between the case without stress inhibition, in which the rate of proliferation is taken as defined in Eq. (5), and the stress-inhibited case using Eq. (6) is put in evidence. It can be observed that, in the case without stress inhibition, the maximum value of the displacement amounts at 3.1 mm; moreover, such a value is not uniform around the tumour and along the three cutting planes: in the $\text {XZ}$-plane, for instance, the maximum displacement is about 2.9 mm. This is a consequence of the patient-specific anisotropy included in the model thanks to the diffusion and permeability tensors: the presence of fibres influences the movement of fluid and nutrients diffusion, which in turn affect the growth, leading to a displacement around the tumour mass which is greater along certain directions. The second row of Fig. 4 shows instead the displacement magnitude in the stress-inhibited case, i.e. when the proliferation term (6) is chosen with parameters $\delta _1 = 0.8$ and $\delta _2 = 10^{-4}$ MPa. It can be noticed that compression strongly inhibits the growth of the tumour, reducing therefore the amount of deformation around its placement; in particular, the maximum displacement is about 1.7 mm, which is almost a half of the one attained in the case without stress inhibition. This result highlights once more the importance of having a model which is able to include mechanical features of tumour growth, both to evaluate the impact of the mass on the healthy tissue and to correctly predict tumour evolution.

Moreover, the cellular proliferation inside the tumour region leads to an increase in the volumetric fraction of the solid phase, as shown in the first row of Fig. 5. After a month and a half of growth without stress inhibition, $\phi _{\mathrm{s}}$ has almost reached the saturation value of 0.85 inside the tumour domain, and it starts to substantially increase also in the surrounding healthy region, due to the compression exerted by the growing mass. On the other hand, if growth becomes sensitive to compressive stresses, the value achieved by the solid fraction is much smaller after the same period of simulation and the changes in volume fraction around the tumour are slightly perceivable. This is consistent with the fact that stress is slowing down tumour proliferation, as observed also in biological experiments (Cheng et al. 2009; Helmlinger et al. 1997; Montel et al. 2012; Delarue et al. 2014).

Concerning some other relevant variables of the model, in the first row of Fig. 6 we show the final values of the fluid pressure *p*, which is negative inside the cancer region, since the fluid is consumed during the uncontrolled cellular proliferation. In the second row of Fig. 6 we plot the value of $\Sigma =-\frac{1}{3}{{\,\mathrm{tr}\,}}(\mathbb {T}_{\mathrm{s}})$, that coincides with the trace of the volumetric solid Cauchy stress, recalling Eq. (23), and it is a stress measure of the compression used in our model to account for stress inhibition of growth. As expected, it is positive inside the tumour, meaning that there is a compression in this area, and negative in zones around the tumour boundary, where the tissue is in traction. The existence of gradients of compression and tension, moving from the tumour towards the surrounding brain tissue is confirmed by biological tests combined with a simple finite element model (Seano et al. 2019; Nia et al. 2017).

Finally, as regards the concentration of nutrients (not shown in the figures), it is almost maintained at the physiological value of 1 in the whole healthy region of the brain, while it substantially decreases inside the tumour, where proliferating neoplastic cells are consuming the nutrients faster than they are supplied.

In addition to the  plots representing the relevant variables on the three-dimensional brain domain, in Fig. 7 we report the line plots along three representative rays originating from the tumour centre and lying in different orthogonal planes. This allows to evaluate spatial evolutions of the variables: it can be seen that the displacement magnitude presents a peak at the tumour boundary and then vanishes as we move away from the cancer domain. Moreover, as already shown before, the solid volume fraction $\phi _{\mathrm{s}}$ displays a non-monotonic behaviour along some rays, due to the fact that the solid phase is growing and compressing the surrounding healthy tissue. Instead, the pressure increases when moving from the tumour centre to the healthy tissue, while the bulk solid stress decreases, coherently with the observation that the maximum compression is experienced inside the cancer proliferation domain.

To compare our results with biological data, we computed the tumour volume evolution for three different values of its elastic parameters: results are shown in Fig. 8a. It can be seen that there is an initial stage where the tumour volume grows approximately linearly: then, growth starts to slow down due to saturation. In detail, when the tumour is ten times stiffer than the healthy tissue, the final volume after 45 days amounts at 3.5 $\hbox {cm}^3$. Moreover, we observe a volume doubling time (VDT) of about 25 days and a specific growth rate (SGR, defined as $(\ln 2)/\text {VDT}$) of 2.8$\%$ per day. These results are indicative of a very fast growth and are in the range of experimental data by Stensjøen et al. (2015), who reported a median VDT of 29.8 days, and by Ellingson et al. (2016) where a median VDT of 21.1 days (with average of $41.0\,\pm \,28.2$ days) is found. Additionally, the SGR ranges from a median of 1.2–2.2$\%$ per day in Stensjøen et al. (2015) to an average of $5.9\, \pm \,2.0$% per day in Ellingson et al. (2016). We remark however that these quantities are usually obtained in medical assays by assuming that growth is exponential, which in some cases might be an oversimplification.

Moreover, even if the tumour in our simulations is highly non-spherical due to anisotropy as discussed before, we computed an average velocity of radial expansion (VRE) to make a comparison with other experimental results. In detail, starting from the tumour volume, we computed its equivalent radius considering it as a sphere and evaluated the expansion velocity along the radial coordinate. Our simulations suggest an average VRE of approximately $v_r \approx 18.4$ mm/year, which is biologically feasible (Stensjøen et al. 2015; Ellingson et al. 2016; Wang et al. 2009; Swanson et al. 2008) even if there is a high clinical variability from patient to patient and in some cases the VRE is even greater. In particular, growth is faster in the first period, when the tumour is still localized and the cell volume fraction is far from the saturation limit. Instead, if we consider a softer tumour, which is only four times stiffer than the surrounding brain tissue, its growth is significantly slower: over the same time span of 45 days, the final cancer volume is about 2.2 $\hbox {cm}^3$, with a relative change in volume of 49%. In this case, the average VRE amounts at $v_r \approx 8$ mm/year, indicative of a slower growth. In the case in which the mechanical parameters inside the tumour region are equal to the ones of the healthy tissue (Nia et al. 2017; Svensson et al. 2022), it can be seen that volume growth is very slow compared to the other situations: the final volume of the mass amounts at 1.7 $\hbox {cm}^3$ and the relative volume change is less than 15%. For each choice of the mechanical parameters, in Fig. 8a we report as dashed lines the corresponding simulation with stress inhibition of growth, setting $\delta _1 = 0.8$ and $\delta _2 = 10^{-4}$ MPa. As expected, the sensitivity of volume growth to compressive stresses increases with the difference in mechanical parameters between the tumour and the host tissue. Overall, the results underline the importance of accounting for the mechanical features of tumour growth, since stiffer cancer masses are more effective in displacing the surrounding healthy tissue and can progress faster.

Furthermore, we compare our model outcomes with experimental data provided in Nia et al. (2017), Seano et al. (2019), Stylianopoulos et al. (2013). In particular, the profiles of the Cauchy stress component $T_{\mathrm{zz}}$ across the tumour diameter and the surrounding tissue, reported in Fig. 8b are in qualitative agreement with the experimental results and finite element estimates provided in Nia et al. (2017). It can be observed that the stress has a peak near the interface between the tumour and the healthy tissue, where tension accumulates. Then, there is a high compression zone inside the tumour, with a slight decrease at the tumour centre. Moreover, if the tumour is ten times stiffer than the host tissue, the modulus of the considered component of the Cauchy stress is significantly higher compared with the case in which the two tissues are mechanically equivalent. In spite of this, the tension and compression values for both $T_{\mathrm{zz}}$ and $\Sigma$ predicted by our model are overall higher when we consider the mechanical parameters of the tumour ten times greater than the ones of the healthy tissue. On the other hand, if we take equal material parameters we obtain results both qualitatively and quantitatively comparable with the ones reported in Nia et al. (2017), Seano et al. (2019), where stresses between $-\,0.1$ and 0.1 kPa are recorded for tumours on mice. These discrepancies may be due to the fact that the stress values are highly dependent on the material model and on the chosen constitutive characterization, and most experimental and computational models, including (Nia et al. 2017; Seano et al. 2019), are based on linear elasticity, while we employ a nonlinear elastic framework. Moreover, in our case we deal with a tumour that has a radius of about 7 mm, which is almost twice as much as the one used in the experimental setup of Nia et al. (2017). Since it has been shown that solid stress increases with tumour radius (Nia et al. 2017), our stress results may be feasible from a biological viewpoint, though further investigation about stress for in vivo tumours is needed as well as an accurate estimation of the mechanical parameters and tumour stiffness. We also find that the tumour solid stress predicted by our model is within the range of residual stresses estimated for cancer spheroids in Stylianopoulos et al. (2013), namely between 1.3 and 13.3 kPa, though these results are not brain specific. In addition, solid stress values are again shown to be higher in modulus in the tumour interior, where there is a consistent amount of compression that slows down the growth of the cancer. Notwithstanding Stylianopoulos et al. (2013) reported that the interstitial fluid pressure increases inside the tumour bulk as a consequence of vascular collapse (not modelled in our framework), our predictions suggest that the pressure is decreased, in accordance with other works using mixture models (Giverso et al. 2015; Giverso and Preziosi 2019). Moreover, in the present work we did not take into account the possibility of reduced perfusion due to cancer growth, which is an effect often reported in the literature (Seano et al. 2019; Stylianopoulos et al. 2013), but it would be interesting to consider it in future research.

Finally, we investigated the role of cancer cell sensitivity to stress inhibition in the progression of the disease. To do so, in Fig. 9 we compare the evolution of the tumour volume for different grades of stress inhibition (regulated by the parameters $\delta _1$ and $\delta _2$), with respect to the case without stress inhibition. In particular, in Fig. 9a we show the volume evolution of the cancer in the case without stress inhibition and in three stress-inhibited cases, varying both the parameters $\delta _1$ and $\delta _2$. Specifically, if we increase the impact of compression by decreasing $\delta _2$ while keeping $\delta _1 = 0.8$ fixed, the volume growth becomes consistently slower and reduces the velocity of cancer expansion. This can be also seen in Fig. 9b, where the three-dimensional configuration of the tumour is shown at the initial time instant and at $t=45$ days, for the case without stress inhibition and for a strongly stress-inhibited case. An evaluation of the velocities of radial expansion yields $v_r = 15.1$ mm/year if $\delta _1 = 0.8$, $\delta _2 = 10^{-3}$ MPa and $v_r = 11.2$ mm/year if $\delta _1 = 0.8$, $\delta _2 = 10^{-4}$ MPa. Instead, keeping $\delta _2 = 10^{-4}$ MPa fixed and increasing $\delta _1$ also leads to a slower growth, even if the volume reduction due to inhibition is smaller.

### Modification of DTI data

Lastly, we show some results related to the DTI data modification due to tumour growth: indeed, the expansion of the mass and the induced displacement alter the fibre tracts in the surroundings, leading to changes in diffusion and preferential directions. To quantify these changes, we recall that, given $\lambda _1> \lambda _2 > \lambda _3$ the descending order eigenvalues of the diffusion tensor, the fractional anisotropy (FA) is a scalar parameter defined by:

43 $$\begin{aligned} {\text {FA}} := \sqrt{\frac{1}{2} \frac{(\lambda _1 - \lambda _2)^2 + (\lambda _2 - \lambda _3)^2 + (\lambda _1 - \lambda _3)^2}{\lambda _1^2 + \lambda _2^2 + \lambda _3^2}}. \end{aligned}$$

A fractional anisotropy of 0 identifies an isotropic medium, where the eigenvalues are all coincident and the diffusion ellipsoid is actually a sphere, with no preferential direction. Instead, a FA value of 1 indicates the existence of a totally preferred direction, making diffusion to occur only along one of the eigenvectors. In order to provide an estimate of how the diffusion tensor is changed in time as a consequence of the tumour-induced deformation, in Fig. 10 we report the difference $\Delta {\mathrm{FA}} = {\mathrm{FA}}_f - {\mathrm{FA}}_i$ of fractional anisotropy between the final and initial diffusion tensors. It can be noted that, in the region surrounding the growing cancer, there is an increase in diffusive anisotropy up to 60%. Variations in FA are also non-uniform in the tumour area, highlighting zones which are significantly affected by changes in anisotropy and others which instead maintain their initial preferential directions. It is also worth to observe that most of the tumour bulk displays no change in FA with respect to the initial value, computed from medical images. Indeed, DTI data extracted from patients are often altered by the tumour, which displaces or even destroys the fibres as it grows, reducing anisotropy inside the tumour bulk, as pointed out in other works (Swan et al. 2018). Since at the beginning of the simulations the tumour has already a size of some millimetres, the most significant changes in anisotropy happen around the tumour domain, where the cancer mass dislocates the surrounding white matter fibres and the displacements are higher.

Since the fractional anisotropy only gives a scalar measure related to the eigenvalues, we also investigated the variation in the eigenvector direction $\mathbf{e} _1^0$ associated with the greatest eigenvalue $\lambda _1$ of the diffusion tensor $\mathbb {D}_0$. To do so, first of all we computed in each mesh cell the direction of the eigenvector $\mathbf{e} _1^f$ associated with the greatest eigenvalue of the diffusion tensor $\mathbb {D}$ at the final time step. In fact, thanks to the modification of the initial tensor $\mathbb {D}_0$, we can keep track of the preferential directions of diffusion, which may vary in each cell due to tumour growth and subsequent deformation. Then, we calculated the value of

$$\begin{aligned} s := 1-|\mathbf{e }_{1}^{0}\cdot \mathbf{e }_{1}^{f}| \, , \end{aligned}$$

that is a measure related to the scalar product between the initial and final eigenvectors. In particular, $s = 0$ denotes zones where the eigenvector does not change as a consequence of the deformation, while $s=1$ identifies regions with the greatest modifications in the direction of $\mathbf{e }_1^0$ due to tumour growth. As shown in Fig. 11, the greatest variations occur in cells located at the border of the tumour region and in the surrounding healthy area. However, the scalar index *s* does not provide details about the change in orientation of the eigenvectors. Therefore, we expressed the eigenvectors $\mathbf{e} _1^0$ and $\mathbf{e }_1^f$ using spherical coordinates $(r,\theta ,\varphi )$, where $r>0$ is the distance from the origin, $\theta \in (-\pi ,\pi ]$ is the azimuthal angle and $\varphi \in (0,\pi ]$ is the polar angle, so that $\mathbf{e }_1^0 = (r^0,\theta ^0,\varphi ^0)$ and $\mathbf{e }_1^f = (r^f,\theta ^f,\varphi ^f)$, and we calculated the absolute differences $|\Delta \theta | = |\theta ^f - \theta ^0|$ and $|\Delta \varphi | = |\varphi ^f - \varphi ^0|$ between the angles $\theta$ and $\varphi$, respectively, at final and initial time instants. Since we are interested in the preferential axis of diffusion and not in its orientation, we identified azimuthal angles and polar angles differing by multiples of $\pi$ and rescaled the angles variation between 0 and $\pi /2$. These variations were only computed in anisotropic regions, where $\mathbb {D}$ is not spherical and therefore it is meaningful to evaluate changes in the eigenvector associated with the greatest eigenvalue. Results for $|\Delta \theta |$ and $|\Delta \varphi |$ are shown in Fig. 11: there are regions, both inside and outside the tumour domain, in which the two angles defining the spherical coordinates of $\mathbf{e} _1^0$ vary, leading therefore to changes in the preferential direction of nutrients diffusion, in qualitative agreement with medical observations (Bouwen et al. 2018). Finally, we remark that the results shown in Figs. 10 and 11 have been obtained after a postprocessing, by elaborating differences between initial and final data on each cell of the mesh.

## Conclusions

Mechanical compression is a common abnormality of brain tumours that has been shown to be responsible for the severe neurological defects of brain cancer patients and to represent a negative prognostic factor (Gamburg et al. 2000; Kalli et al. 2019). To refine previous mathematical descriptions of brain tumour growth and account for this mechanical impact, we proposed a model that explicitly features hyperelastic deformations of brain tissue and incorporates medical imaging data coming from DTI and MRI. Using the well-established framework of Continuum Mechanics, we described the brain and the cancer mass as saturated biphasic mixtures, comprising a solid and a fluid phase, which are both relevant in a hydrated soft tissue like the brain. This enables us to evaluate deformations and stresses caused by the proliferation of tumour cells, as well as the displacement induced on the surrounding healthy tissue. In particular, thanks to the multiphase approach, the model is able to distinguish the stress contribution associated with the fluid from the one associated with the solid mass, and therefore it could be useful in understanding the biological implications and the extent of the so-called mass-effect (Chauhan et al. 2014; Goriely et al. 2015; Jain et al. 2014; Seano et al. 2019; Kalli et al. 2019). The mechanical description included in the present model allows to account for growth inhibition due to excessive compression, thanks to the definition of a proliferation term embedding a proper stress measure. Moreover, by numerically computing the deformation field, it is possible to modify the diffusion and permeability tensors, taking into account the displacement of the brain tissue. This is a clear advantage for simulations of brain tumour growth, since it allows to consider changes in the DTI data without the need of repeating the clinical screening exams multiple times.

Our results and outcomes show the possibility of using our model as a proof-of-concept to make a step forward in the realistic description of cancer in the brain. Specifically, we highlighted the unnatural displacement caused by the tumour, as well as the increase in volume depending on both the mechanical properties of the tumour itself and on the amount of stress inhibition. We were also able to quantify changes in the fractional anisotropy of the tissue and in the orientation of preferential directions.

The model therefore suggests the relevance of a realistic mechanical description of brain cancer growth. However, there are still some limitations in our approach which should be addressed in future improvements. For instance, it should be appropriate to overcome the choice of a continuous and regularized indicator function for the tumour region. Even though the infiltration of tumours like GBM into the surrounding tissues justifies its description using an interface that is not totally sharp, for solid tumours it would be probably more realistic to implement a discontinuous separation between the diseased and healthy areas. In addition, the inclusion of an anisotropic growth tensor should be evaluated: in this work we consider the influence of anisotropy by defining patient-specific diffusion and permeability tensors, but it would be interesting to treat growth distortions as anisotropic as well. The problem of reconstructing DTI coefficients and therefore realistic anisotropy inside the tumour region is also not trivial, since medical imaging data are often isotropic inside the cancer bulk because of fibres disruption. Thus, the modelling of anisotropy changes may require further research efforts, by using for instance a reflected DTI approximation (Swan et al. 2018) or solving an inverse problem to quantify the relevant DTI parameters in zones where the imaging is altered by the cancer. The damage of white matter fibres due to the growth of the tumour could also be taken into account, by introducing an explicit description of the amount of healthy fibres in the tissue.

Then, future works should also be dedicated to the accurate estimation of all the relevant parameters of the model. Specifically, the present framework can be easily extended to incorporate patient-specific mechanical data, obtained by MRE. Indeed, thanks to the improvements attained in the last years to optimize high-resolution multifrequency MRE acquisition protocols and preprocessing tools, it is now possible to have 3D MRE data with 2 mm isotropic resolution and strong repeatability. Anyhow, the commonly used processing tools for parameters identification in MRE are based on inversion analysis for wave propagation in a linear viscoelastic medium (Murphy et al. 2019; Fehlner et al. 2017), therefore future studies are still needed to reconstruct the inherently nonlinear mechanical response of brain tissues. In addition to the mechanical parameters, other constants like the densities of the solid and fluid phase in the brain could be evaluated more precisely to improve the multiphase framework, in accordance with recent findings (Ehlers et al. 2022). Finally, the model should be validated on a real set of data, to test its predictive capability.

Further developments of the proposed model might include also the simulation of therapies, like radiotherapy effects, and surgical removal of the tumour mass. With regard to the latter, the rearrangement of tissue after resection strongly depends on the mechanical forces and on the tumour-induced reorganization of healthy tissues and fibres in the region around the cancer, that has been included in the presented mechanical perspective.

## Supplementary Information

Below is the link to the electronic supplementary material.

Supplementary material 1 (PDF 1758 kb)
