# Supplementary material for: Coupling solid and fluid stresses with brain tumour growth and white matter tract deformations in a neuroimaging-informed model
Source: Biomech Model Mechanobiol. 2022 Jul 30;21(5):1483–509. doi: 10.1007/s10237-022-01602-4 (PMC9626445; doi:10.1007/s10237-022-01602-4)
Supplement: Supplementary file 1 — Supplementary material 1 (PDF 1758 kb) [file 10237_2022_1602_MOESM1_ESM.pdf]

Coupling solid and fluid stresses with brain tumour growth and white matter tract deformations in a neuroimaging-informed model

Supplementary Information

G. Lucci[1] A. Agosti[2] P. Ciarletta[3] C. Giverso[1*]

[1]Department of Mathematical Sciences "G.L. Lagrange", Politecnico di Torino, Corso Duca degli Abruzzi 24, 10129 Turin, Italy
[2]Department of Mathematics, University of Pavia, Via Ferrata 5, 27100 Pavia, Italy
[3]MOX-Politecnico di Milano, Piazza Leonardo da Vinci 23, 20133 Milan, Italy

April 29, 2022

*Corresponding author. E-Mail address: `chiara.giverso@polito.it`

Supplementary Notes

A1. Simulations near the skull and boundary conditions

In some cases, brain tumours may appear near the brain boundary, leading to the generation of high stresses and extremely asymmetric growth patterns due to the rigid constraint that the skull imposes. To investigate such a situation using our mathematical and computational model, we performed two set of simulations with different boundary conditions: the results are shown in Figs. S1-S5.

It is worth to remark that, when we wish to simulate cancer growth near the skull, the boundary conditions become very relevant and need to be evaluated carefully. In the main simulations presented in the article, the tumour was placed in the same zone where it was recorded by MRI and the following Dirichlet boundary conditions were applied:

$$\mathbf{u}_s = \mathbf{0} \quad \text{on} \quad \partial\Omega^*, \forall\, t \in (0,T)\,, \tag{S.1a}$$
$$p = 0 \quad \text{on} \quad \partial\Omega^*, \forall\, t \in (0,T)\,, \tag{S.1b}$$
$$c_n = 1 \quad \text{on} \quad \partial\Omega^*, \forall\, t \in (0,T)\,. \tag{S.1c}$$

In particular, the first boundary condition stems from the rigidity of the skull, which cannot be displaced, and it represents the most natural constraint on the solid deformation. Instead, conditions (S.1b)-(S.1c) correspond to fixing both the pressure and the nutrients concentration on the boundary of the brain. They represent a fairly reasonable assumption when the tumour is placed sufficiently far from the skull and its growth does not have a direct impact on the values of the variables at the boundary, which may then be fixed at reference values. Instead, if the cancer mass is positioned in close proximity to the skull, zero-flux Neumann boundary conditions might be appropriate. In fact, it is enough to impose that the fluid and the chemicals cannot flow out of the boundary, without necessarily forcing them to attain specific values. Therefore, in the simulations near the skull, we chose to use both the Dirichlet boundary conditions (S.1) and the following set of conditions:

$$\mathbf{u}_s = \mathbf{0} \quad \text{on} \quad \partial\Omega^*, \forall\, t \in (0,T)\,, \tag{S.2a}$$
$$\mathbb{K}^* \,\text{Grad}\, p \cdot \mathbf{N} = 0 \quad \text{on} \quad \partial\Omega^*, \forall\, t \in (0,T)\,, \tag{S.2b}$$
$$\mathbb{D}^* \,\text{Grad}\, c_n \cdot \mathbf{N} = 0 \quad \text{on} \quad \partial\Omega^*, \forall\, t \in (0,T)\,, \tag{S.2c}$$

where $\mathbf{N}$ is the outer normal vector to the boundary $\partial\Omega^*$, $\mathbb{K}^* = J_s\mathbb{F}^{-1}\mathbb{K}\mathbb{F}^{-\mathrm{T}}$ and $\mathbb{D}^* = J_s\mathbb{F}^{-1}\mathbb{D}\mathbb{F}^{-\mathrm{T}}$. In this case, we keep the fixed null displacement and impose zero normal flux conditions on p and c_n. In Figs S1-S5 we compare the results using the two different sets of boundary conditions (S.1) and (S.2).

As expected, the presence of the fixed skull generates a pronounced asymmetry in the tumour growth pattern, that can be seen clearly in the displacement and solid volume fraction plots, especially in the case of Dirichlet boundary conditions (S.1). Moreover, when the zero-flux conditions (S.2c) on c_n are imposed, the concentration of nutrients decreases more around the tumour region, since there is no supply coming from the boundary as in the Dirichlet case.

Supplementary Figures

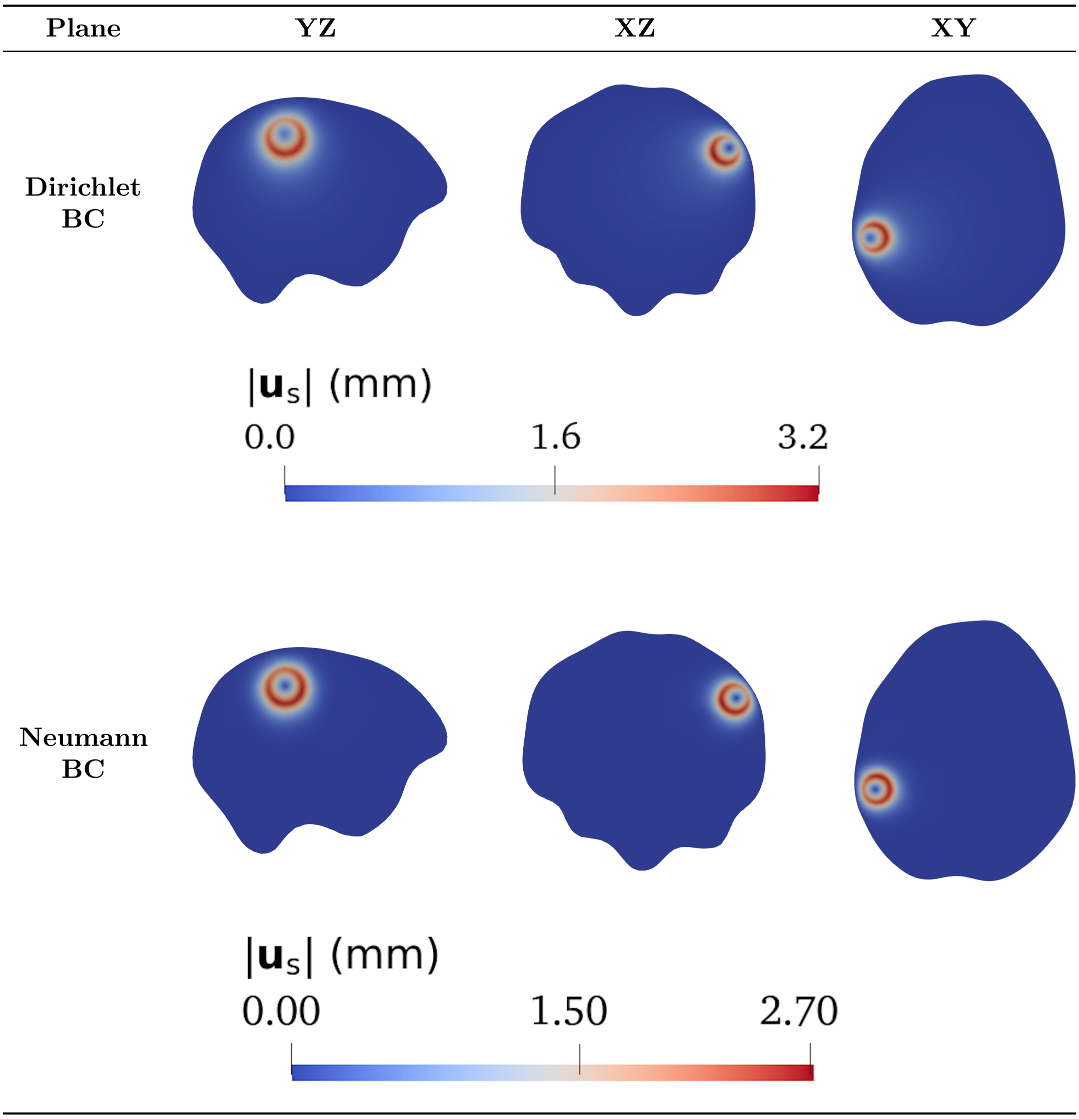

Figure S1: Comparison between the displacement magnitude $\|\mathbf{u}_s\|$ after $t = 35$ days of tumour growth near the skull, clipped along a sagittal (first column), an axial (second column) and coronal (third column) plane centered within the tumour. In the first row, the case with Dirichlet boundary conditions as from Eqs. (S.1) is reported, while the second row shows the case with zero-flux boundary conditions for the pressure and concentration of nutrients, as stated in Eqs. (S.2).

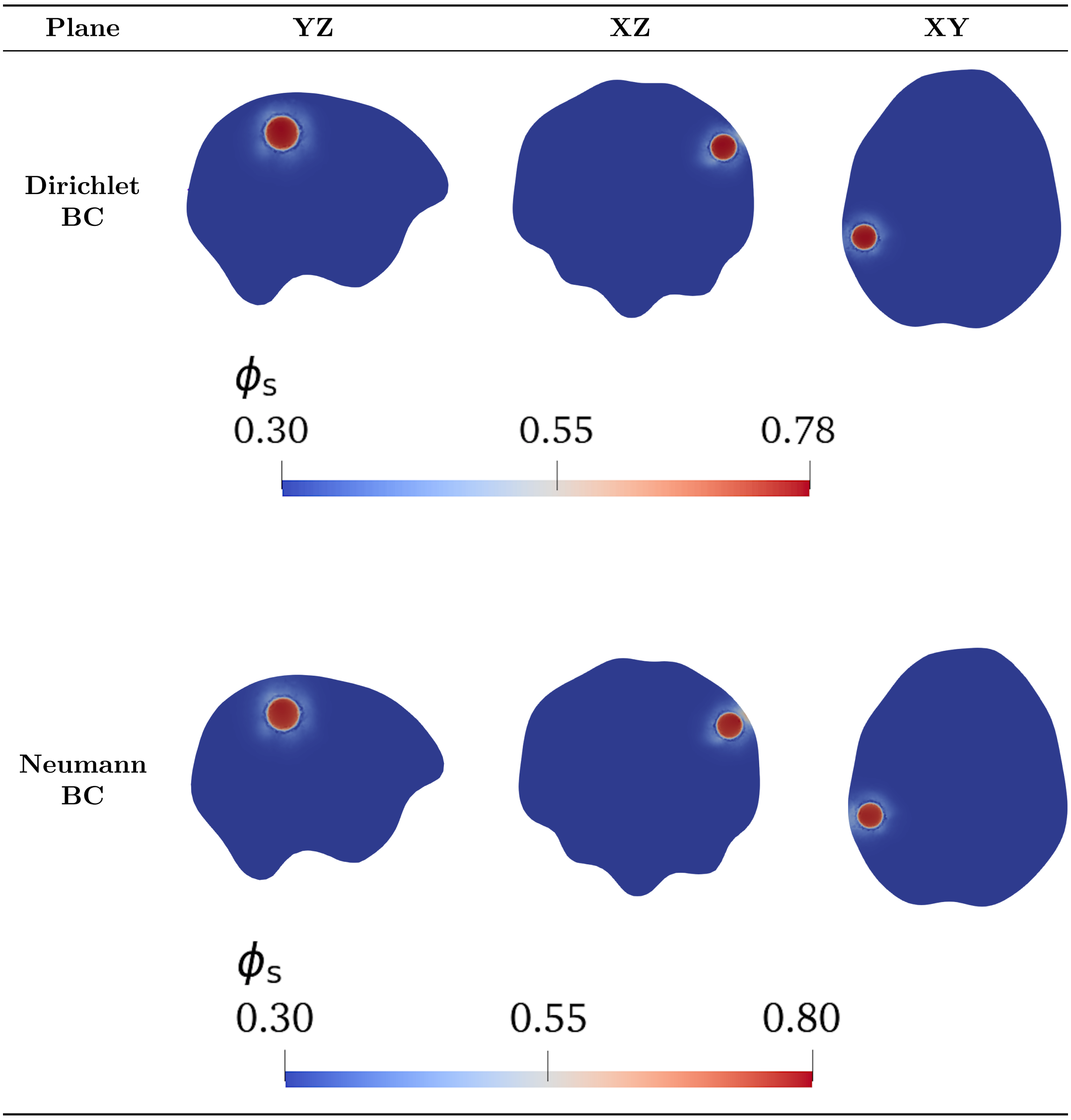

Figure S2: Comparison between the solid volume fraction ϕ_s after $t = 35$ days of tumour growth near the skull, clipped along a sagittal (first column), an axial (second column) and coronal (third column) plane centered within the tumour.In the first row, the case with Dirichlet boundary conditions as from Eqs. (S.1) is reported, while the second row shows the case with zero-flux boundary conditions for the pressure and concentration of nutrients, as stated in Eqs. (S.2).

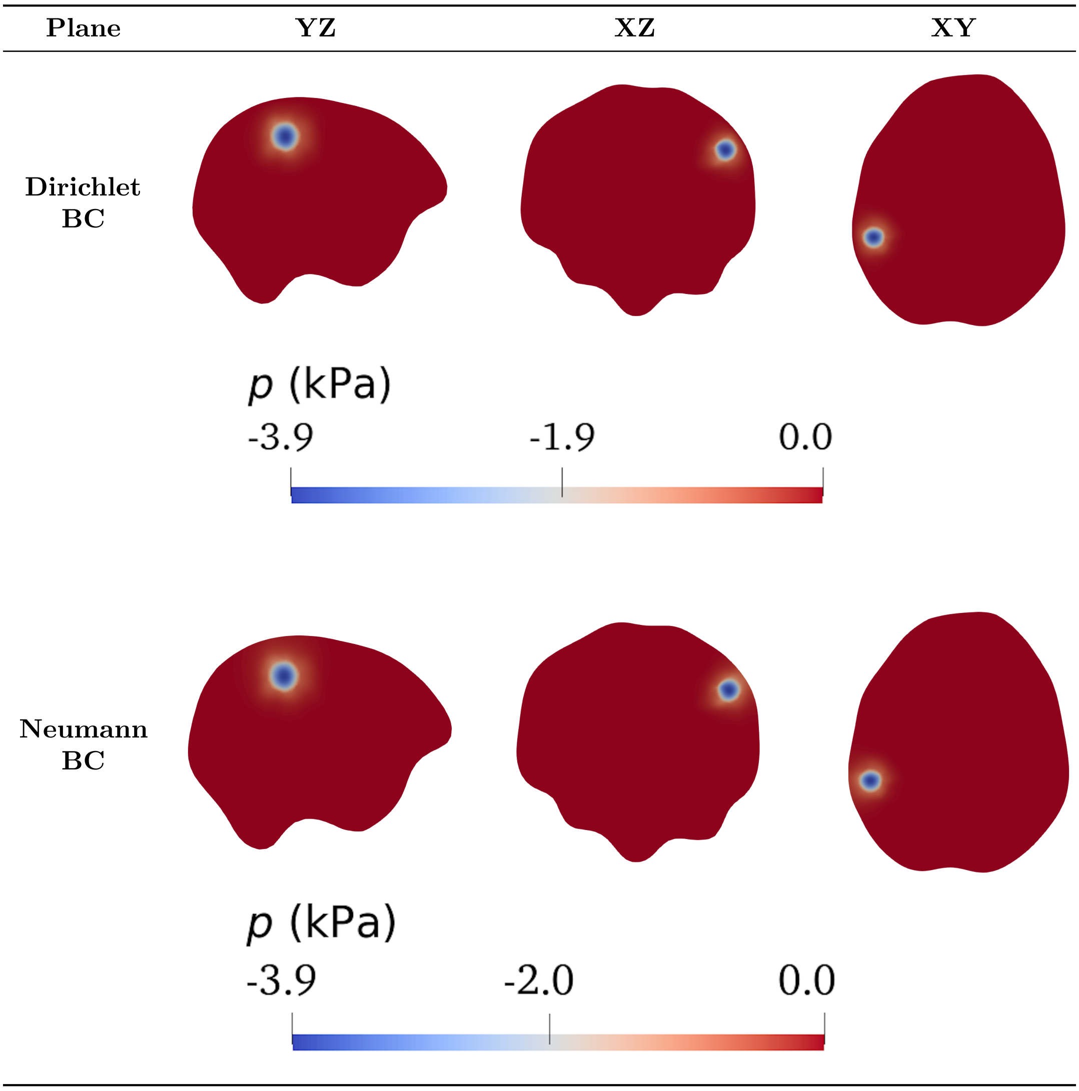

Figure S3: Comparison between the pressure p after $t = 35$ days of tumour growth near the skull, clipped along a sagittal (first column), an axial (second column) and coronal (third column) plane centered within the tumour. In the first row, the case with Dirichlet boundary conditions as from Eqs. (S.1) is reported, while the second row shows the case with zero-flux boundary conditions for the pressure and concentration of nutrients, as stated in Eqs. (S.2).

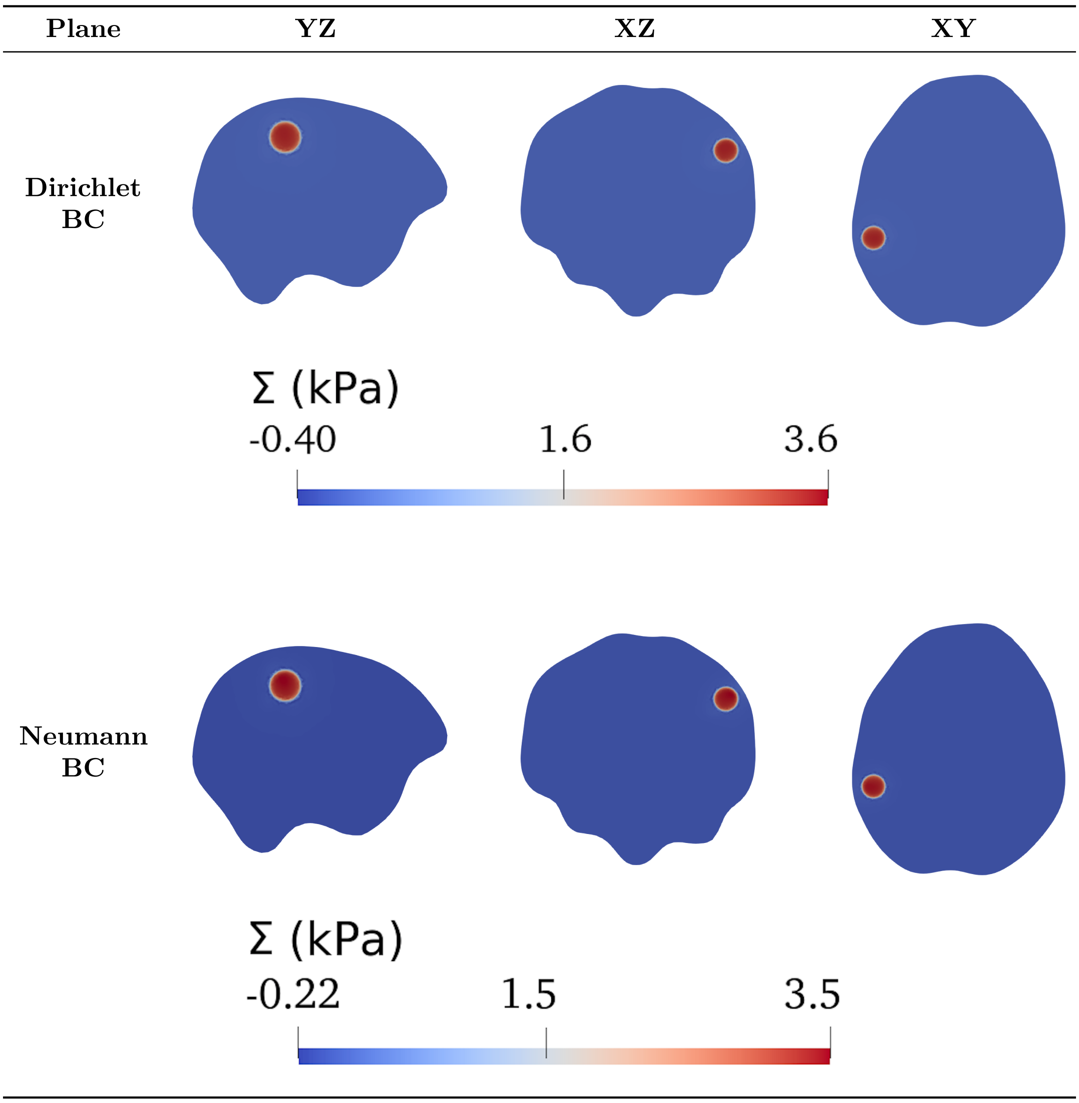

Figure S4: Comparison between the bulk solid stress Σ after $t = 35$ days of tumour growth near the skull, clipped along a sagittal (first column), an axial (second column) and coronal (third column) plane centered within the tumour. In the first row, the case with Dirichlet boundary conditions as from Eqs. (S.1) is reported, while the second row shows the case with zero-flux boundary conditions for the pressure and concentration of nutrients, as stated in Eqs. (S.2).

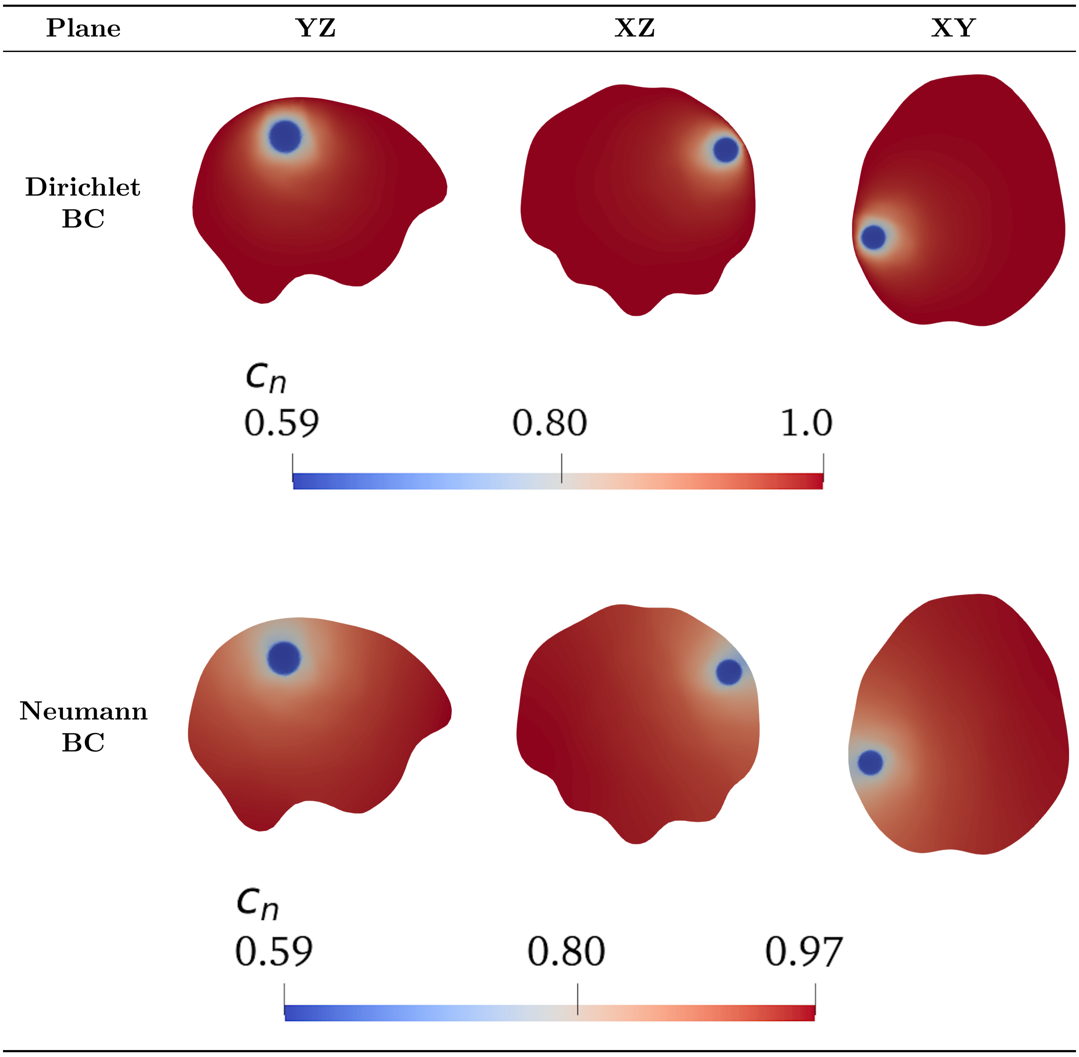

Figure S5: Comparison between the concentration of nutrients c_n after $t = 35$ days of tumour growth in the brain, clipped along a sagittal (first column), an axial (second column) and coronal (third column) plane centered within the tumour. In the first row, the case with Dirichlet boundary conditions as from Eqs. (S.1) is reported, while the second row shows the case with zero-flux boundary conditions for the pressure and concentration of nutrients, as stated in Eqs. (S.2).
